# Epigenetic changes mediated by polycomb repressive complex 2 and E2a are associated with drug resistance in a mouse model of lymphoma

**DOI:** 10.1186/s13073-016-0305-0

**Published:** 2016-05-04

**Authors:** Colin Flinders, Larry Lam, Liudmilla Rubbi, Roberto Ferrari, Sorel Fitz-Gibbon, Pao-Yang Chen, Michael Thompson, Heather Christofk, David B Agus, Daniel Ruderman, Parag Mallick, Matteo Pellegrini

**Affiliations:** Department of Biological Chemistry, University of California, Los Angeles, CA 90095 USA; Department of Molecular, Cellular and Developmental Biology, University of California, Los Angeles, CA 90095 USA; Department of Molecular and Medical Pharmacology, University of California, Los Angeles, CA 90095 USA; Department of Medicine, University of Southern California, Los Angeles, CA 90033 USA; Canary Center, Stanford University, Palo Alto, CA 94305 USA; Center for Applied Molecular Medicine, University of Southern California, Los Angeles, CA 90033 USA

**Keywords:** Burkitt’s lymphoma, Mafosfamide, Resistance, Histone, Methylation, Epigenetics

## Abstract

**Background:**

The genetic origins of chemotherapy resistance are well established; however, the role of epigenetics in drug resistance is less well understood. To investigate mechanisms of drug resistance, we performed systematic genetic, epigenetic, and transcriptomic analyses of an alkylating agent-sensitive murine lymphoma cell line and a series of resistant lines derived by drug dose escalation.

**Methods:**

Dose escalation of the alkylating agent mafosfamide was used to create a series of increasingly drug-resistant mouse Burkitt’s lymphoma cell lines. Whole genome sequencing, DNA microarrays, reduced representation bisulfite sequencing, and chromatin immunoprecipitation sequencing were used to identify alterations in DNA sequence, mRNA expression, CpG methylation, and H3K27me3 occupancy, respectively, that were associated with increased resistance.

**Results:**

Our data suggest that acquired resistance cannot be explained by genetic alterations. Based on integration of transcriptional profiles with transcription factor binding data, we hypothesize that resistance is driven by epigenetic plasticity. We observed that the resistant cells had H3K27me3 and DNA methylation profiles distinct from those of the parental lines. Moreover, we observed DNA methylation changes in the promoters of genes regulated by E2a and members of the polycomb repressor complex 2 (PRC2) and differentially expressed genes were enriched for targets of E2a. The integrative analysis considering H3K27me3 further supported a role for PRC2 in mediating resistance. By integrating our results with data from the Immunological Genome Project (Immgen.org), we showed that these transcriptional changes track the B-cell maturation axis.

**Conclusions:**

Our data suggest a novel mechanism of drug resistance in which E2a and PRC2 drive changes in the B-cell epigenome; these alterations attenuate alkylating agent treatment-induced apoptosis.

**Electronic supplementary material:**

The online version of this article (doi:10.1186/s13073-016-0305-0) contains supplementary material, which is available to authorized users.

## Background

Many anticancer therapies lose effectiveness over time as tumors acquire resistance. Despite significant study [1], acquired resistance remains a major obstacle to improving remission rates and achieving prolonged disease-free survival. A number of explanations for resistance have been proposed, including the presence of cancer stem cells [2] and mutations that confer drug resistance [3].

Although Burkitt’s lymphoma is extremely aggressive [4], 90–95 % of children receiving the standard-of-care therapy, a combination treatment of rituximab, cyclophosphamide, doxorubicin, vincristine, and prednisone, enter complete remission. Treatment of adults is not as successful [5], in part due to acquired resistance. In both children and adults, salvage treatment has a poor success rate, with only one-third of children and very few adults obtaining positive outcomes from salvage therapy.

The mechanisms underlying acquisition of resistance in Burkitt’s lymphoma are only partially understood. Numerous genetic mechanisms have been hypothesized, including up-regulation of expression of proteins involved in drug efflux, such as the ATP-binding cassette transporter family, cyclophosphamide inactivation through aldehyde dehydrogenase up-regulation, increased expression of DNA repair enzymes, and deregulation of apoptosis through the loss of *Tp53* [1]. Genetic mutations are unable to explain cases of acquired resistance that arise rapidly or that reverse in response to a drug holiday [6, 7].

Alterations in histone modifications and DNA methylation that lead to an altered transcriptional program have been proposed to lead to acquired drug resistance in B-cell lymphoma [8, 9]. Recent work in an in vitro model of Burkitt’s lymphoma has shown that treatment with the DNA methylation inhibitor 5-azacytidine reactivates expression of *Id2*, which encodes a repressor of translocated Myc, resulting in the inhibition of proliferation [10]. Similarly, treatment of lymphoma cell lines with the histone deacetylase inhibitor suberoylanilide hydroxamic acid has been shown to re-sensitize lymphoma cell lines to various therapeutic agents [11].

Studies of clinical specimens have revealed that tumors are both genetically and epigenetically heterogeneous [8, 12]. The role of genetic heterogeneity within tumors and its effect on treatment response and outcome has been extensively studied, but less is known about how epigenetic heterogeneity impacts disease progression and clinical outcome. Previous studies have shown that drug treatment generates selective pressure on heterogeneous populations that leads to the enrichment of specific genetically distinct subpopulations [13, 14]. These subpopulations can ultimately become the dominant population in a tumor, resulting in resistance to the therapeutic agent. It is possible that similar mechanisms of selection act at the epigenetic level. Recent research in prostate cancer has documented the inherent heterogeneity of DNA methylation in patient tumor samples [15], though the selection of epigenetically distinct subpopulations has yet to be shown.

Here we used dose escalation with mafosfamide on parental *Eμ-Myc Cdkn2a*^-/-^ non-Hodgkin’s B-cell lymphoma cells to generate a series of drug-resistant cell lines. We then investigated the mechanism by which these cells acquired resistance. In our experiment, parental cells were cultured with increasing doses of mafosfamide, from 100 nM to 4 μM, over 5 weeks. At four steps in the dose escalation, resistant clones were isolated and molecularly profiled. Whole-genome sequencing of the parental and resistant cell lines did not reveal any genetic alterations that might explain the acquired resistance. However, analyses of transcriptomic and DNA methylation profiling suggested that the acquired resistance is associated with genes and promoters targeted by transcription factor E2a and by members of the polycomb repressive complex 2 (PRC2), Suz12 and Pcl2. An integrative analysis considering histone H3 lysine 27 trimethylation (H3K27me3) further supported a role for PRC2 in mediating resistance. Comparison of the transcriptomic data from resistant lines and from B cells at different developmental stages [16] suggested that resistance is associated with epigenetic changes that are found along the B-cell maturation axis. E2a is a master regulator of B-cell development in mice. An analysis of human diffuse B-cell methylation data revealed that the methylation status of genes regulated by the human ortholog of E2a, TCF3, is associated with treatment failure. Thus, our study implicates epigenetic factors in evolution of acquired resistance.

## Methods

### Creation of resistant *Eμ-Myc Cdkn2a*^-/-^ lines

The parental *Eμ-Myc Cdkn2a*^-/-^ lymphoma line was generated from a C57BL/6 J mouse as described in Schmitt et al. [17]. Lymphoma cells from these mice were cultured in vitro to generate the cell line. Resistant strains were generated from this parental line via dose escalation from 100 nM to 4 μM of mafosfamide (Cell Signaling Technology) over a 34-day period.

### Cell viability and cell cycle analysis

Cell viability was measured using the Perkin-Elmer Operetta platform with 2.5 μM Draq5 (Abcam) for nuclear detection and 5 μg/mL of propidium iodide (Sigma-Aldrich, St. Louis, MO, USA) to detect dead cells. For cell cycle analysis, cells were fixed in ethanol and placed in solution with propidium iodide. Cells were gated for G0/G1, S, and M phase on a Beckman LSRII.

### Genome sequencing

Genomes of cell lines were sequenced to a minimum of 8× average coverage using Illumina HiSeq 2000 sequencers. The reads were aligned to the mm9 (MGSCv37) *Mus musculus* reference genome using BWA version 0.6.2-r126 (backtrack) [18] with default parameters. Duplicate reads were removed using PICARD version 1.85(1345) with default parameters (Additional file 1). The whole-genome sequencing data are available via the Sequence Read Archive under accession number SRP071753.

### Oligonucleotide microarray analysis

Oligonucleotide microarray analysis was carried out using Affymetrix GeneChip Mouse Gene ST 1.0. The resulting data are publically available via Gene Expression Omnibus accession GSE60342. Data were quantified and processed with robust multi-array averaging using the justRMA function of the 1.40.0 affy R package [19]. Expression values were log_2_ transformed for further downstream analysis. Probe sets were annotated using the Affymetrix MoGene-1_0-st-v1.na33.2.mm9.probeset.csv file. We selected the top 1000 probe sets ranked by their covariance to identify differentially regulated genes (Additional file 1).

### Transcription factor analysis

Targets for 64 murine transcription factors were identified from ChIPBase (http://deepbase.sysu.edu.cn/chipbase, downloaded August 1, 2013) [20] and limited to genes with binding events within 5 kilobases (kb) of transcriptional start sites. To identify potential upstream regulators, we identified the overlap of chromatin immunoprecipitation-sequencing (ChIP-seq) data with predicted transcription factor targets and used a one-sided Fisher’s exact test to determine significance.

### ChIP-seq

Chromatin was immunoprecipitated as described previously [21]. Briefly, cells were grown to 50 % confluency. Formaldehyde was added for 10 min at room temperature and 100 μl of the lysate (5 × 10^6^ cells) was used for each immunoprecipitation with anti-H3K27me3 (Active Motive, catalogue number 39155). Libraries were sequenced using an Illumina HiSeq 2000 to obtain 50-bp-long reads.

Peaks were called by comparing counts in the immunoprecipitated libraries with input libraries in windows tiling the genome using Poisson statistics as previously described [21]. Combinatorial clustering of data was achieved by determining significant enrichment for the histone mark in each condition within 5 kb upstream of transcription start sites (at least three 50-bp bins with *p* < 1.0e-6). A binary distribution was created based on a promoter being enriched (1) or not enriched (0) and a combinatorial matrix was created with all possible combinations across all conditions. H3K27me3 data were plotted based on the combinatorial clustering and visualized by a Cluster 3.0-generated CDT file loaded on Java-Tree view to produce a heat map. The ChIP-seq datasets are publically available via Gene Expression Omnibus accession GSE78939.

### Bisulfite sequencing

Reduced representation bisulfite (RRBS) libraries were generated following the standard RRBS protocol [22]. The genome was digested with the methylation-insensitive restriction enzyme MspI and fragments from 100 to 300 bases were selected. The fragments were ligated with Illumina adaptors, denatured, and treated with sodium bisulfite. The libraries were sequenced using Illumina HiSeq 2000 sequencers. The reads were aligned using the bisulfite aligner BS Seeker2 [23] to determine where fragments uniquely mapped allowing for three mismatches to the reference genome (mouse mm9). The RRBS datasets are publically available via Gene Expression Omnibus accession GSE78939.

### DNA methylation analysis

To identify DNA methylation changes correlated with resistance, we computed RRBS fragment CpG methylation levels and calculated the covariance between the fragment methylation score and sample order (ordered from least to most resistant; Additional file 1).

### Principal component analysis

Principal component analysis of expression profiles was performed by applying the R *prcomp* function with the scaled option to the expression microarray values of the resistant cell lines and B cells at different stages of development (NCBI Gene Expression Omnibus accession GSE15907). See Additional file 1 for samples used.

## Results

### Generation of resistant lines and assessment of cell cycle characteristics

**Fig. 1:**
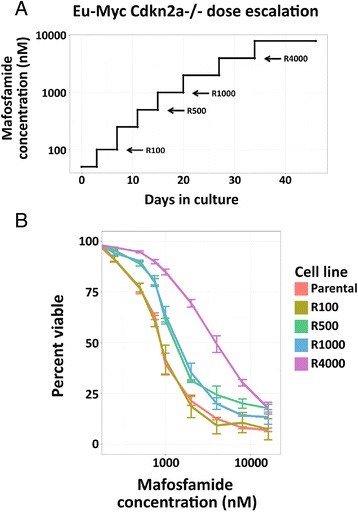
Dose escalation of *Eμ-Myc Cdkn2a* ^-/-^ cells and resistance to mafosfamide. **a** *Eμ-Myc Cdkn2a* ^-/-^ cells were cultured with increasing doses of mafosfamide. After 10, 18, 23, and 37 days, corresponding to cells cultured in 100, 500, 1000, and 4000 nM of mafosfamide, surviving cells were harvested. **b** The percentage viability of the resistant cells was measured after 24-h exposure to mafosfamide

To investigate the factors driving acquisition of resistance to chemotherapy, we employed a widely used cell line derived from an *Eμ-Myc* mouse model of Burkitt’s lymphoma [17]. We refer to this line as our “parental” line. This model has two genetic alterations: (1) a translocation in the *c-Myc* oncogene that causes its expression to be controlled by an immunoglobulin heavy chain enhancer, thus restricting its expression to B cell lineage cells; and (2) a deletion in *Cdkn2a* that recapitulates a common mutation seen in human tumors [24]. *Eμ-Myc Cdkn2a*^-/-^ mice develop lymphomagenesis with highly invasive tumors with apoptotic defects [17]. Resistant lines were generated by gradually exposing the parental line to increasing concentrations from 100 nM to 4 μM of mafosfamide (an in vitro active form of cyclophosphamide) in cell culture (Fig. 1a, b; Additional file 1: Figure S1). Four resistant sub-clones were isolated during the 5-week period of dose escalation (R100, R500, R1000, and R4000) for subsequent analysis. We refer to these as the “resistant” lines. The cycling rate of our parental and resistant lines in the absence of mafosfamide was very similar (Additional file 1: Table S1, Figure S2), suggesting that the resistance is not associated with large changes in cell cycle phases.

Mafosfamide treatment led to a greater percentage of apoptotic cells in the parental line relative to the resistant lines (Fig. 1b; Additional file 1: Table S1, Figures S1 and S2). In all lines, non-apoptotic cells showed an equivalent cell cycle delay as assessed by the decrease in percentage of cells in G1 and a concurrent increase in G2 percentage upon treatment. As all the lines showed approximately the same amount of cell cycle delay upon treatment in the non-apoptotic fraction, resistance is most likely not a result of an increased cell cycle delay.

### Resistance is unlikely to have arisen by genetic mechanisms

Given the four-fold difference in drug sensitivity between the parental and resistant cell lines (parental half maximal effective concentration, EC_50_, 857 ± 68 nM; R4000 EC_50_ 3446 ± 138 nM, mean ± standard error of the mean), we initially hypothesized that acquired resistance was mediated by genetic variants that were enriched in the population over the course of the dose escalation. To identify possible resistance-associated variants, we performed whole-genome sequencing and identified single-nucleotide variants (SNVs) that showed an increase in allele frequency that correlated with increased resistance. Twenty-three SNVs displayed this pattern (Additional file 1: Figure S3). Of these, 13 had variant alleles in only one sample (the most resistant line) and manual inspection of read alignments showed that the SNVs all occurred solely at the ends of sequencing reads or in repetitive regions (Additional file 1: Table S2) and were, therefore, likely sequencing artifacts. To validate that these were in fact false positives, we performed Sanger sequencing on regions surrounding five of the potential SNVs. No mutations were observed using this sequencing technique (Additional file 1: Figure S4). Additionally, no novel large structural variations or potential copy number variations were found in any of the lines. Together, these data suggest that genetic alterations are not the principal explanation for the acquired resistance.

### Alterations in gene expression of mafosfamide-resistant cell lines

To determine whether the decreased sensitivity to mafosfamide observed in the resistant lines was associated with altered transcription, we measured the changes in gene expression for the parental and each resistant line. A subset of these gene expression changes were confirmed by quantitative PCR (Additional file 1: Figure S5). We expected to identify genes known to be involved in drug metabolism, transport of mustard alkylating agents, and DNA repair [25, 26]. We found that the expression of most genes involved in these processes did not increase significantly in our resistant lines, suggesting that none of these processes are likely to play a major role in mediating resistance (Additional file 1: Figure S6).

**Fig. 2:**
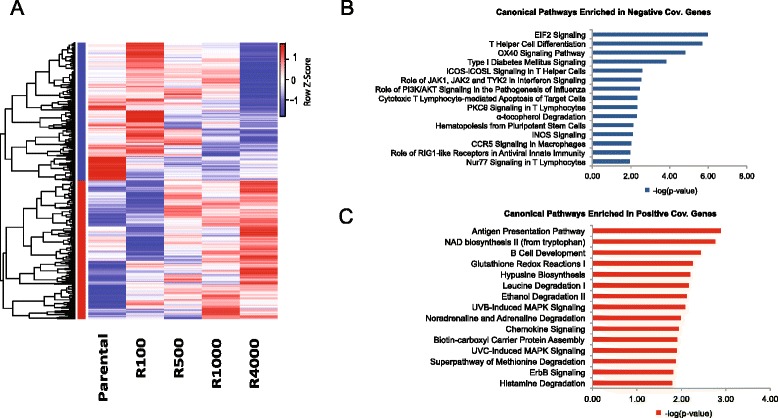
Microarray expression profile of untreated cell lines. **a** Heat map of the probe sets. Positive covariance with increasing resistance to mafosfamide is indicated by the *red cluster*; negative covariance with resistance is indicated by the *blue cluster*. **b** The top 15 pathways enriched in negative covariance genes. **c** The top 15 pathways enriched in positive covariance genes

To identify transcripts with expression patterns that were perturbed with increasing resistance, we computed the covariance of the expression of each gene with the EC_50_ of the resistant cell line. The 1000 genes with highest and lowest covariance were clustered (Fig. 2a). Pathway analysis of those genes that showed a positive covariance with resistance (i.e., an increase in gene expression with increasing resistance) indicated that they encode proteins involved in B-cell maturation and development (Fig. 2b). Genes that showed a negative covariance were enriched for those associated with EIF2-mediated signaling and T helper cell differentiation (Fig. 2c).

### DNA methylation changes with drug resistance

**Fig. 3:**
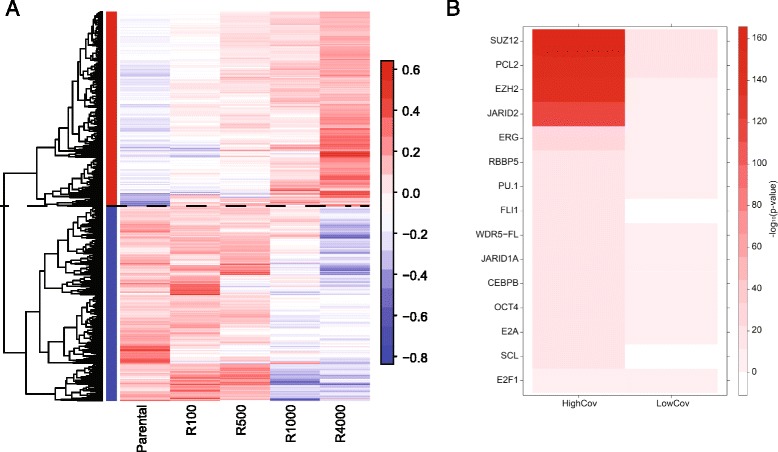
DNA methylation analysis. **a** Heat map of the top and bottom 1000 RRBS fragments based on covariance. **b** Heat map of the –log10(*p*) values for the overlap of high and low covariance genes with transcription factor targets

DNA methylation is a central mechanism of transcriptional regulation and alterations in the methylome have previously been shown to contribute to lymphomagenesis and drug resistance [27–29]. To gain further insights into epigenetic changes that occurred during the acquisition of drug resistance, we measured DNA methylation using reduced representation bisulfite sequencing (RRBS) of the parental and resistant lines. This approach allowed us to quantify the methylation of approximately one million CpGs. To identify methylation sites associated with drug resistance, we computed the covariance between the methylation state of each CpG and the EC_50_ of the resistant cell line. Covarying sites were clustered (Fig. 3a). High covariance RRBS fragments had lower levels of gene expression compared with overall expression based on all annotated probes, whereas low covariance RRBS fragments had higher levels of gene expression (Additional file 1: Figure S7). The top 1000 high covariance RRBS fragments were located in gene regions that were functionally enriched for DNA binding, transcription factor activity, and cell differentiation (Additional file 1: Table S3).

We next asked whether the genes that were proximal to these sites were associated with specific transcription factors. Each transcription factor from ChIPBase [20] was tested for association with the 1000 genes with the highest and lowest DNA methylation covariance with respect to resistance (Fig. 3b). From this analysis, we identified the transcription factors E2a and PU.1; these transcription factors are involved in B-cell development [30–33]. We also found that members of the polycomb repressive complex 2 (PRC2) Suz12 and Pcl2 were very strongly enriched over positively covarying genes, suggesting an involvement of this complex in mediating epigenetic changes during the acquisition of drug resistance. The expression of PRC2 members was similar in parental and resistant lines; however, *Uty*, which encodes one of three known histone H3 lysine 27 demethylases [34], was down-regulated in the resistant lines, suggesting that Uty may be involved in the epigenome alteration that confers drug resistance (Additional file 1: Figure S8a, b).

### Resistant cell lines show altered H3K27me3 occupancy

**Fig. 4:**
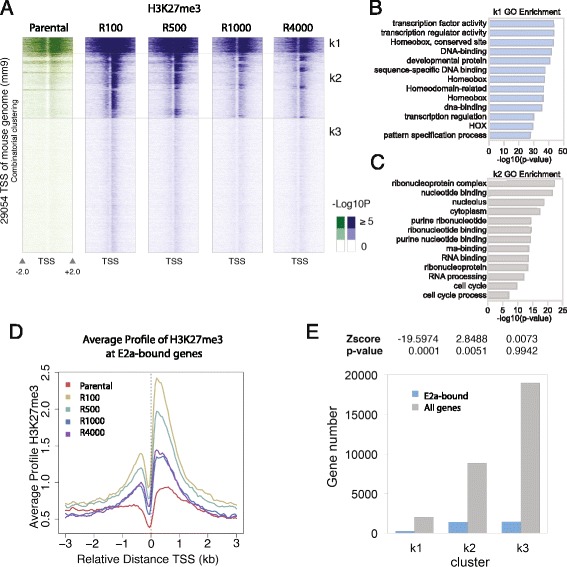
H3K27me3 ChIP-seq analysis. **a** Clusters of promoters based on their H3K27me3 levels in the parental and resistant lines (*n* = 2). *TSS* transcription start site. **b** Group K1 shows enrichment for developmental genes. *GO* gene ontology. **c** Group K2 shows enrichment for cell cycle-regulated genes. **d** Average H2K27me3 levels across E2a-bound genes. **e** Analysis of E2a-bound genes in the three clusters

As PRC2 has been shown to be involved in placement of the H3K27me3 mark [35], we performed ChIP-seq for H3K27me3 in all lines (grown in the absence of mafosfamide) to verify the involvement of PRC2 in the transition to drug resistance. We found widespread acquisition of H3K27me3 at the transcriptional start sites of a large group of genes in the least resistant (R100, R500) lines (Fig. 4a). These effects appeared to be stable across biological replicates, as seen by the high correlation between replicate profiles (Additional file 1: Table S4). However, the lines with increased resistance (R1000, R4000) had decreased levels of H3K27me3 with respect to the parental line at many loci.

As most H3K27me3 peaks are near the transcription start sites, we analyzed promoters by means of combinatorial clustering (described in “Methods”) and identified three major groups of genes: those with a high level of H3K27me3 throughout the time course (K1), those with rapid and widespread H3K27me3 acquisition followed by a gradual decrease (K2), and those with little to no H3K27me3 (K3). Functional analysis of these groups showed enrichment for developmentally regulated genes in K1 and cell cycle-regulated genes in K2 (Fig. 4b, c). The level of H3K27me3 across all genes bound by E2a revealed that the average profile followed a similar pattern of change to that of cluster K2 (Fig. 4d). Furthermore, E2a-bound genes were enriched in cluster K2 and were depleted from K1 (Fig. 4e). These results suggest that H3K27me3 occupancy may regulate expression of E2a-bound genes.

Previously published literature shows a linkage between Ezh2-mediated H3K27 methylation and DNA methylation. Ezh2 is the catalytic subunit of PRC2. Analysis of our RRBS data revealed that DNA methylation levels proximal to K2 genes increased between the parental and resistant cell lines (Additional file 1: Figure S9a); we did not observe an increase in DNA methylation in genes in K1 or K3 subsets compared with parental levels (Additional file 1: Figure S9b, c). Additionally, we found a significant overlap between the high covariance RRBS fragments and the K1 and K2 clusters (Additional file 1: Figure S10). These results suggest a linkage between H3K27me3 and DNA methylation at loci with altered expression during acquisition of resistance.

### Principal component analysis of basal gene expression indicates alterations in B-cell maturation

The observation that changes in expression, DNA methylation, and H3K27me3 levels in the resistant lines involve genes that encode regulators of B-cell development (e.g., E2a and PU.1) led us to hypothesize that gradual dose escalation results in epigenetic changes associated with a B-cell maturation axis. To identify the transcriptional profile characteristic of the B-cell maturation axis, we used the Immunological Genome Project (Immgen.org) dataset [36], which contains expression data collected from B-cell progenitors, cells at multiple stages during maturation, and mature B cells.

**Fig. 5:**
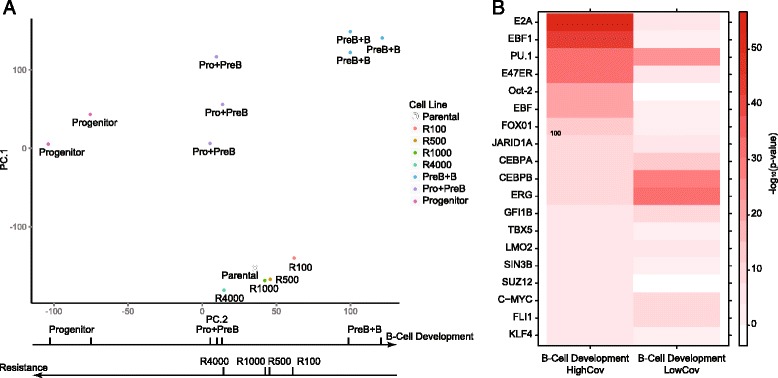
B-cell development principal component analysis. **a** Scatter plot of the principal components of the Immunological Genome Project data and the data on resistant lines. **b** Heat map of the –log10(*p*) values for the overlap of high and low covariance genes with transcription factor target genes

To associate our expression data with the B-cell developmental axis, we performed a combined principle component analysis of gene expression data obtained from parental and resistant cell lines and B cells at different stages of development gathered from the Immgen dataset. We found that the second principal component, PC.2, captures the developmental state of B cells (Fig. 5a) with less differentiated progenitor cells toward the negative direction of the PC.2 axis and the more mature states in the positive. Our four resistant lines are ordered by their differentiation, with the least resistant line more differentiated than the most resistant line. This result suggests that the transcriptomes of our cells vary monotonically along this B-cell developmental axis. Additionally, functional annotation of the top principal component genes showed enrichment for the B-cell receptor signaling pathway in the top 1000 PC.2 genes (Additional file 1: Table S5).

To identify factors associated with this B-cell developmental axis, we identified genes that had a positive correlation with development (i.e., an increase in expression during differentiation). We then used ChIPBase data to identify factors with targets enriched within this list. Genes positively correlated with development were often targets of E2a and PU.1 (Fig. 5b).

### Differential methylation status of E2a target genes is associated with treatment failure in patients with aggressive B-cell lymphoma

To confirm that E2a mediates resistance development, we sought to determine the methylation status of genes bound by TCF3, the human ortholog of E2a, in a clinically relevant dataset. We obtained DNA methylation data from diffuse large B-cell lymphoma (DLBCL) patients collected from the Cancer Genome Atlas project. DLBCL differs from Burkitt’s lymphoma but is the most similar lymphoma for which we were able to access data on clinical samples.

Of 19 DLBCL samples available, 14 corresponded to patients who were tumor-free after their initial treatment course and five were from patients who experienced disease progression. For all methylation sites, we computed the Kolmogorov–Smirnov statistic and an associated *p* value between these two groups. From 482,421 total CG sites on the Illumina 450 K array, 5541 had a Kolmogorov–Smirnov test with *p* < 0.05. We linked CpG sites to TCF3 binding regions if both the CpG and binding region occurred within the gene body or within 10 kb of the gene’s transcription start site. We then identified the intersection of genes that had both evidence of TCF3 binding and at least two CpG sites with significant methylation changes between the two patient populations. From a total of 17,744 genes used in the linking calculations, we derived Table 1, which shows a strong enrichment of TCF3-bound genes associated with differentially methylated sites (*p* value ≤ 0.0001). This analysis suggests that TCF3-bound genes are differentially methylated and are associated with treatment failure in a lymphoma subtype.

**Table 1:** TCF3 target gene methylation

	TCF3 target	Not TCF3 target
Differential methylation	208	80
No differential methylation	5086	12,370

DNA methylation data obtained from The Cancer Genome Atlas shows an increase in differential methylation in TCF3 target genes in patients with DLBCL who showed stable or progressive disease compared with individuals who had complete response

To further test the clinical significance of differential methylation, we compared lists compiled from our gene expression and DNA methylation analyses with a 1458-gene signature of DLBCL survival obtained from the Precog data set [37]. Fifty-eight genes from the top 449 high expression covariance gene list and an additional 58 genes from the top 597 high methylation covariance significantly overlapped with the 1458 genes associated with DLBCL survival (*p* = 0.0001 and *p* = 0.0006, respectively; Additional file 1: Figure S11a, b). Furthermore, those genes that overlap between our H3K27me3 cluster K1 and the high covariance RRBS fragments (Additional file 1: Figure S10) similarly significantly overlapped with the 1458 genes associated with DLBCL survival (Additional file 1: Figure S11c).

## Discussion

By treating *Eμ-Myc Cdkn2a*^-/-^ cells with escalating doses of mafosfamide, we developed four cell lines with increasing resistance to the drug. We hypothesized that if a mutation provided a proliferative or survival advantage, then the frequency of that mutation would increase over time in culture. We observed no verifiable mutations that displayed this pattern.

Reasoning that measuring gene expression changes across cell lines might reveal the resistance mechanism, we calculated the covariance of gene expression with respect to increasing resistance. Pathway enrichment analysis showed an enrichment for genes encoding proteins involved in B-cell development. Given the role that DNA methylation plays in B-cell lineage commitment [28, 38], we characterized the DNA methylome of our resistant lines. Analysis of DNA methylation changes suggested that expression of genes regulated by the histone methyltransferase complex PRC2 were altered in the most resistant lines.

PRC2 plays a critical role in B-cell development by repressing genes necessary for hematopoietic differentiation through the addition of tri-methylation to lysine 27 of histone H3. During hematopoietic stem cell differentiation, this repressive mark is lost from genes that commit hematopoietic stem cells to differentiation [30, 31, 39]. Numerous studies have shown that overexpression of, or activating mutations in, PRC2 components, particularly Ezh2, contribute to proliferation and lymphomagenesis in DLBCL patients [40, 41]. To evaluate how the presence of the H3K27me3 mark is correlated with resistance, we performed ChIP-seq on H3K27me3 in the parental and resistant cell lines. A large fraction of promoters in the resistant lines showed rapid and widespread H3K27me3 acquisition followed by a gradual reduction. This pattern of rapid H3K27 methylation followed by demethylation has been reported in patients: loss of H3K27me3 is a predictor of poor outcome [42]. These results suggest that PRC2 is rapidly activated by the DNA damage induced by mafosfamide, which is consistent with previous literature suggesting the PRC2 is targeted to sites of DNA damage [43, 44]. Furthermore, these results suggest that this initial response is attenuated as cells become adapted to higher doses of mafosfamide and the most resistant cells have an H3K27me3 state that is similar to that of the parental cells.

The observation that H3K27me3 is rapidly gained but then lost suggests that it may not be the only mechanism leading to resistance in our setting. The removal of H3K27me3 at many loci may lead to its replacement with DNA methylation, a more permanent mark. It has been suggested that histone methylation by PRC2 may recruit Dnmt3l, an inactive homolog of DNA methyltransferase, resulting in the inhibition of CpG methylation [32]. The gradual removal of H3K27me3 could lead to the replacement of Dnmt3l by its active counterparts, Dnmt3a and Dnmt3b, resulting in an increase in DNA methylation. In agreement with a potential coupling between PRC2 and DNA methyltransferases, we observed that genes that gained H3K27me3 early and then lost this mark (cluster K2) showed a gradual increase in DNA methylation. Thus, we conclude that the addition of H3K27me3 to these loci results in the acquisition of DNA methylation, leading to a stable, drug-resistant epigenome.

Our analyses also indicate the involvement of B-cell developmental regulators in the epigenetic transition to drug resistance. Among these regulators, which include E2a and PU.1, we believe that E2a plays a central role. Clustering of the H3K27me3 ChIP-seq data showed that E2a is strongly associated with genes that gain and subsequently lose H3K27me3 that also gradually gain DNA methylation. Genes with significant levels of differential DNA methylation were also enriched in those bound by E2a. *E2a* encodes two proteins, E12 and E47, that are known to be master regulators involved in the process of B-cell lineage commitment [33]. Principle component analysis of gene expression from the resistant lines and from various stages of B-cell development indicated that the expression of E2a target genes is strongly correlated with B-cell maturation states. In addition, the *E2a* locus is repressed early in B-cell development but it becomes transcriptionally active during B-cell commitment [45]. The central role of E2a-mediated regulation of gene expression in Burkitt’s lymphoma is made evident by the fact that it is the fifth-most mutated gene in patients with Burkitt’s lymphoma and all mutations affect the DNA binding domain [46]. Inhibition of *E2a* expression using small interfering RNA results in lower levels of *Cdkna1* (*p21*) and higher *PUMA* expression, impairment of cell cycle arrest, and increased Tp53-dependent apoptosis [47]. Our data suggest that E2a may be playing a critical role in mediating the resistance phenotype as it appears to be a key regulator of the response to mafosfamide.

Taken together, our results suggest that PRC2 and known regulators of B-cell development induce epigenetic changes in genes encoding key hematopoietic developmental and pluripotency genes that result in the resistant phenotype. Upon treatment with mafosfamide, the cells we studied underwent rapid and widespread acquisition of the repressive mark H3K27me3. This methylation is coupled to changes in the activity of B-cell developmental regulators, such as E2a, ultimately resulting in changes in transcription of genes involved in B-cell development. As the cells became more resistant, the H3K27me3 response was attenuated and replaced by the more permanent mark 5-methylcytosine. We speculate that these changes led the *Eμ-Myc* cell lines to epigenetically transition along the B-cell developmental axis, leading to a suppression of the apoptotic response upon exposure to mafosfamide. Evidence for this transition is provided by our integrative analysis of Immgen data that captures a principal component of the B-cell developmental axis. As the cells became more resistant to mafosfamide, they appear to move backwards along this axis. The diminished apoptotic response of cells with stem-like characteristics compared with that of differentiated cells has been described in many other systems and may thus represent a general mechanism for the acquisition of drug resistance [48, 49].

Finally, we have shown that similar mechanisms may also occur in human lymphomas. Our analysis of samples from patients with diffuse large B-cell lymphoma suggests that there are epigenetic differences between patients who remain disease-free versus those who have disease progression. Moreover, the sites of differential methylation are significantly associated with genes that are bound by Tcf3, the human ortholog of E2a. Thus, our murine model may capture epigenetic resistance mechanisms that are also present in human disease, suggesting that epigenetic plasticity impairs therapeutic regimens in the clinic.

## Conclusions

Our results indicate that resistance to DNA alkylating agents in B-cell lymphoma is associated with alterations in both CpG methylation and H3K27me3 but not with genetic changes. We found that repressive epigenetic markers are preferentially altered in the promoter regions of genes bound by the B-cell development regulator E2a*,* suggesting that this transcription factor plays a key role in mediating the resistance phenotype, possibly by suppressing apoptosis*.* We also showed that differential methylation of genes bound by TCF3, the human ortholog of E2a*,* is associated with treatment failure in diffuse large B-cell lymphoma patients.

### Availability of data and materials

The datasets supporting the conclusions of this article are available in the Gene Expression Omibus repository, accession numbers GSE60342, GSE60342, and GSE78939, as well as the Sequence Read Archive, accession number SRP071753.

## Additional file

Additional file 1: Supplementary Results and Methods. All supplementary figures, tables, legends, results, methods, and references are contained in Additional file 1. (ZIP 2461 kb)

## Abbreviations

ChIP-seq: chromatin immunoprecipitation followed by sequencing
DLBCL: diffuse large B-cell lymphoma
EC_50_: half maximal effective concentration
H3K27me3: histone H3 lysine 27 trimethylation
PRC2: polycomb repressive complex 2
RRBS: reduced representation bisulfite sequencing
SNV: single nucleotide variant
