# Supplementary material for: Epigenetic changes mediated by polycomb repressive complex 2 and E2a are associated with drug resistance in a mouse model of lymphoma
Source: Genome Med. 2016 May 4;8:54. doi: 10.1186/s13073-016-0305-0 (PMC4857420; doi:10.1186/s13073-016-0305-0)
Supplement: Additional file 1: — Supplementary Results and Methods. All supplementary figures, tables, legends, results, methods, and references are contained in Additional file 1. (ZIP 2461 kb) [file 13073_2016_305_MOESM1_ESM.zip › 20151117_GB_Supplementary_Methods_Results.docx]

**Supplementary Figures**

**Supplementary Figure 1 - High content screening images**

DRAQ5 and propidium iodide images of the parental and R4000 lines in the presence and absence of 4uM of mafosfamide.

**Supplementary Figure 2 - FACS analysis of cell cycle.**

A) FACS quantification of the parental cells, B) parental cells after treatment, C) untreated 4000 resistant cells, and D) treated 4000 resistant cells. E) Cell cycle analysis of Parental, R500, R1000, and R4000 in the absence and presence of 750nM of mafosfamide.

**Supplementary Figure 3 - Allele frequency of putative mutations.**

Genes identified that show increasing single nucleotide variance (SNV) frequency in relation to increasing resistance among cell lines.

**Supplementary Figure 4 - Sanger sequencing confirmation of putative mutations.**

Sanger sequencing chromatograms and IGV screenshots of putative mutations in genes A) Romo1, B) Ghsr, C) Zfp605, D) Brsk1, and E) Ostn.

**Supplementary Figure 5 - qPCR and microarray gene expression comparison.**

Comparison of gene expression as measured by qPCR and microarray of genes that showed changes in expression between parental and resistant cell lines.

**Supplemental Figure 6 - Gene expression of genes known to be involved in resistance**

Gene expression changes as measured by microarrays of genes known to be involved in nitrogen mustard based chemotherapies. A) Aldehyde dehydrogenase genes. B) Drug efflux genes. C) DNA repair genes.

**Supplementary Figure 7 - Expression Distribution of Genes Associated with High and Low Covariance RRBS Fragments**

The red boxplots reveal the expression distribution of the genes associated with high covariance fragments across the cell lines. The green boxplots reveal the expression distribution of the low covariance fragments across the cell lines. The blue boxplots reveal the global or expression distribution of all gene annotated probes across all the cell lines. The Welch’s t-test p-value was calculated between the high covariance expression distribution and the low covariance expression distribution for each cell line with a p-value < 1.00E-38. The Welch’s t-test p-value was also calculated between the high covariance expression distribution to the expression distribution of all the genes with a p-value < 1.00E-16.

**Supplementary Figure 8 - Gene expression of PRC2 components and H3K27 demethylases**

A) qPCR of components of the PRC2 complex. B) qPCR of H3K27 demethylases.

**Supplementary Figure 9 - Analysis of DNA methylation fragments**

A-C) DNA methylation fragment entropy histogram of groups K1, K2, and K3 respectively.

**Supplementary Figure 10 - Intersection of H3K27Me3 and RRBS Associated Genes**

A) Venn diagram of the genes from the k1 and k2 cluster in the H3K27Me3 data and the genes associated with high and low covariance RRBS fragments. B) The hypergeometric overlap p-values for the intersection of the RRBS covariance gene sets and the k1 or k2 genes.

**Supplementary Figure 11 - Overlap of DLBCL Survival Genes**

1458 Genes associated with DLBCL survival was selected from the Precog dataset with a minimum false discovery rate $\leq$ 0.01. A) 58 genes intersect with the top 449 genes by expression covariance with a hyper geometric test p-value $<$ 0.0001. B) 58 genes intersect with the top 597 by methylation covariance with a hyper geometric test p-value of 0.0006. C) 9 genes that intersect with the K1 cluster and the high covariance RRBS genes also intersect with the 1458 genes associated with DLBCL survival with a hypergeometric overlap p-value = 0.0441

**Supplementary Table 1 - Cell Cycle Analysis**

Cell cycle analysis of parental and resistant lines in the presence or absence of 750 nM mafosfamide.

**Supplementary Table 2 - List of 23 SNVs that show increasing allele frequency with respect to increasing resistance.**

**Supplementary Table 3 - GREAT GO Annotation of High Covariance RRBS Fragments**

The top 1000 high covariance RRBS fragments were submitted to GREAT for association to GO terms with a background of 358,230 mappable RRBS fragments with regions to gene association set to 100kb.

**Supplementary Table 4 - Correlation of H3K27me3 ChIP-seq replicate experiments.**

**Supplementary Table 5 - Functional Annotation of PC.1 and PC.2 Genes**

The genes were ranked by their PC.1 and PC.2 rotations. The top 1000 genes (PC1 or PC2 High) and bottom 1000 genes (PC1 or PC2 Low) were submitted to DAVID for functional annotation. The most significant term is listed from each cluster with a false discovery rate $\leq$ 0.05.

**Supplementary Methods**

**Genome Sequencing**

SNP and INDEL discovery and genotyping were performed across all 8 samples simultaneously using HaplotypeCaller (GATK) [[1](#_ENREF_1)]. Variants were kept if any one of the samples had a genotype quality score greater than 90 yielding 168,263 variant sites, most of which were heterozygous in all lines. Further selection for alleles not present in the parental line, and with either equal or increasing alternate allele frequencies over the dose escalation series of cell lines yielded 23 variants. Breakdancer-max version 1.1.2 and Pindel version 0.2.4t were used to identify larger structural variations [[2](#_ENREF_2), [3](#_ENREF_3)]. CnD version 1.3 was used to identify copy number variations [[4](#_ENREF_4)].

**Covariance of gene expression**

The covariance was determined for each probe set across increasing treatment time or resistance level,

$$Cov(x,t)= \sum_{i}^{n} {( x}_{i}-\bar{x} )(t_{i}-\bar{t} )$$

where $x$is the vector of mean adjusted probe set expression values across samples, and $\bar{x}$ is the mean expression value; $t$is the vector of the treatment or resistance order and $\bar{t}$ is the mean order. Low expression covariance was determined by selecting the bottom 1000 probe sets ranked by their covariance to identify down regulated genes across the time course or resistant cell lines. Hierarchical clustering was performed on the high and low expression covariance probe sets using Euclidean distance of the mean adjusted probe set expression values.

**DNA Methylation Analysis**
To determine the fragment CpG methylation score, we only considered the 1,599,123 CpG sites with a minimum coverage of four reads common to all the samples. The fragment methylation score is the Z score of the CpG methylation levels within the RRBS fragment standardized by the number of CpG sites within the fragment,

$$z= \frac{\bar{X}-\mu}{\frac{\sigma}{\sqrt{N}}}$$

where $\bar{X}$ is the average CG methylation within the fragment, µ is the mean CpG methylation of the sample methylome, σ is the standard deviation of the sample methylome, and N is the number of CpG sites within the fragment.

High fragment methylation covariance was determined by selecting the top 1000 fragments ranked by their covariance. Low fragment methylation covariance was determined by retrieving the bottom 1000 fragments ranked by their covariance. The high and low covariance fragments were annotated using GREAT, restricting associations to genes within 5 kb of each region [[5](#_ENREF_5)].

**Principle Component Analysis**

The B cell developmental samples were from the Immunological Genome Project[[6](#_ENREF_6)]. A subset of the raw Affymetrix Mouse Gene ST 1.0 microarray datasets from the Immunological Genome Project was downloaded from the NCBI GEO data repository (GSE15907). The subset includes B cells sourced from the fetal liver and bone marrow of C57BL/6J mice at different stages of B cell development to include stem cell, lymphoid progenitor, pro B, pre B and immature B cell stages.

**Enrichment of DLBCL survival genes**

Human genes and their association values to human survival were downloaded and extracted from the PRECOG analytical tool[[7](#_ENREF_7)]. 1458 Genes with a false discovery rate of $<$ 0.05 were used in our overlap analysis with genes associated with changes in expression, fragment methylation and H3K27Me3 binding.

**Supplementary References**

1. McKenna A, Hanna M, Banks E, Sivachenko A, Cibulskis K, Kernytsky A, Garimella K, Altshuler D, Gabriel S, Daly M, DePristo MA: **The Genome Analysis Toolkit: a MapReduce framework for analyzing next-generation DNA sequencing data.** *Genome Res* 2010, **20:**1297-1303.

2. Chen K, Wallis JW, McLellan MD, Larson DE, Kalicki JM, Pohl CS, McGrath SD, Wendl MC, Zhang Q, Locke DP, et al: **BreakDancer: an algorithm for high-resolution mapping of genomic structural variation.** *Nat Methods* 2009, **6:**677-681.

3. Ye K, Schulz MH, Long Q, Apweiler R, Ning Z: **Pindel: a pattern growth approach to detect break points of large deletions and medium sized insertions from paired-end short reads.** *Bioinformatics* 2009, **25:**2865-2871.

4. Simpson JT, McIntyre RE, Adams DJ, Durbin R: **Copy number variant detection in inbred strains from short read sequence data.** *Bioinformatics* 2010, **26:**565-567.

5. McLean CY, Bristor D, Hiller M, Clarke SL, Schaar BT, Lowe CB, Wenger AM, Bejerano G: **GREAT improves functional interpretation of cis-regulatory regions.** *Nat Biotechnol* 2010, **28:**495-501.

6. Heng TS, Painter MW: **The Immunological Genome Project: networks of gene expression in immune cells.** *Nat Immunol* 2008, **9:**1091-1094.

7. Gentles AJ, Newman AM, Liu CL, Bratman SV, Feng W, Kim D, Nair VS, Xu Y, Khuong A, Hoang CD, et al: **The prognostic landscape of genes and infiltrating immune cells across human cancers.** *Nat Med* 2015, **21:**938-945.
