# Supplementary material for: Epigenetic changes mediated by polycomb repressive complex 2 and E2a are associated with drug resistance in a mouse model of lymphoma
Source: Genome Med. 2016 May 4;8:54. doi: 10.1186/s13073-016-0305-0 (PMC4857420; doi:10.1186/s13073-016-0305-0)
Supplement: Additional file 1: — Supplementary Results and Methods. All supplementary figures, tables, legends, results, methods, and references are contained in Additional file 1. (ZIP 2461 kb) [file 13073_2016_305_MOESM1_ESM.zip › 20151118-Supplementary_results.pdf]

Supplementary Figure 1

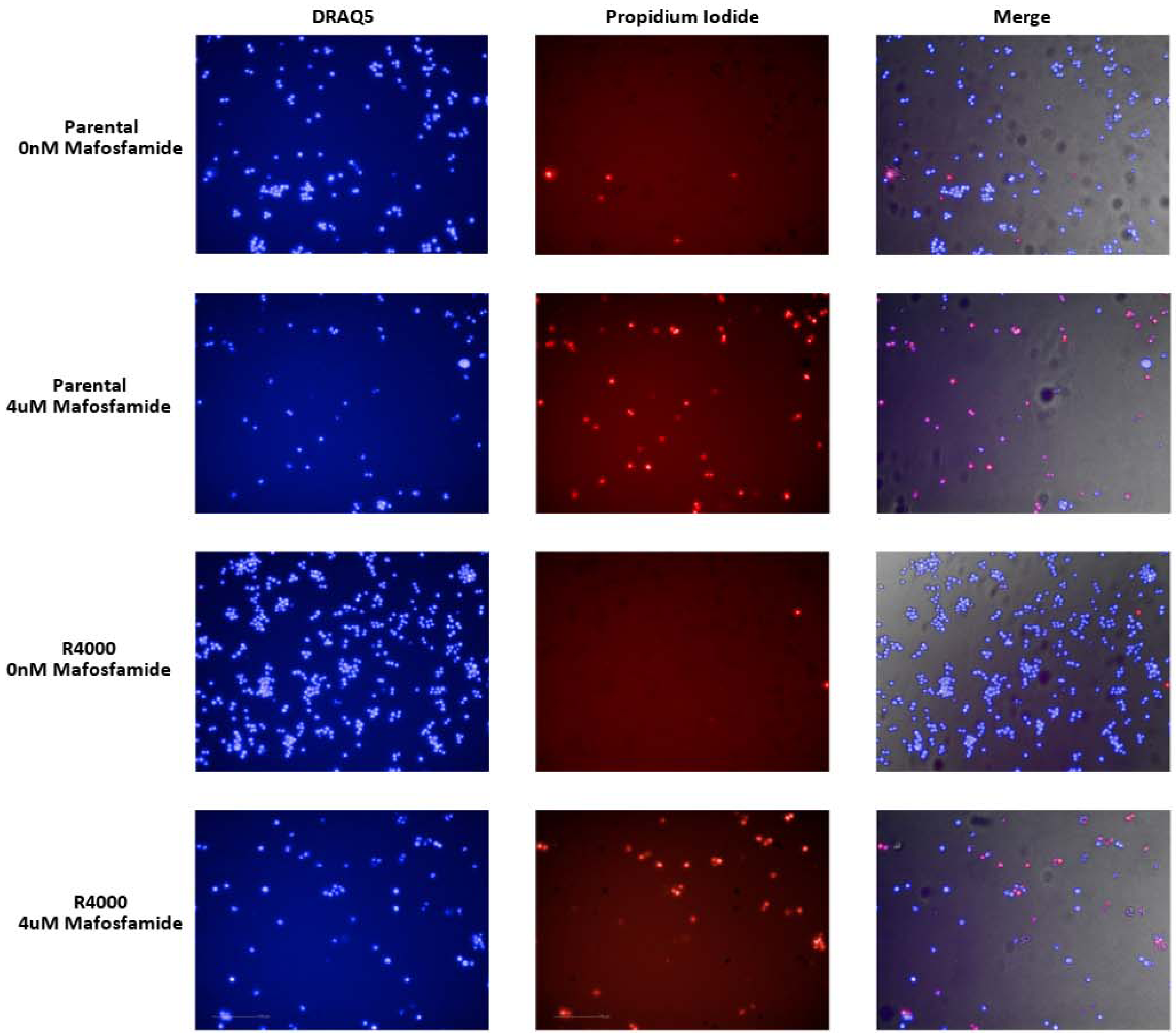

Supplementary Figure 2

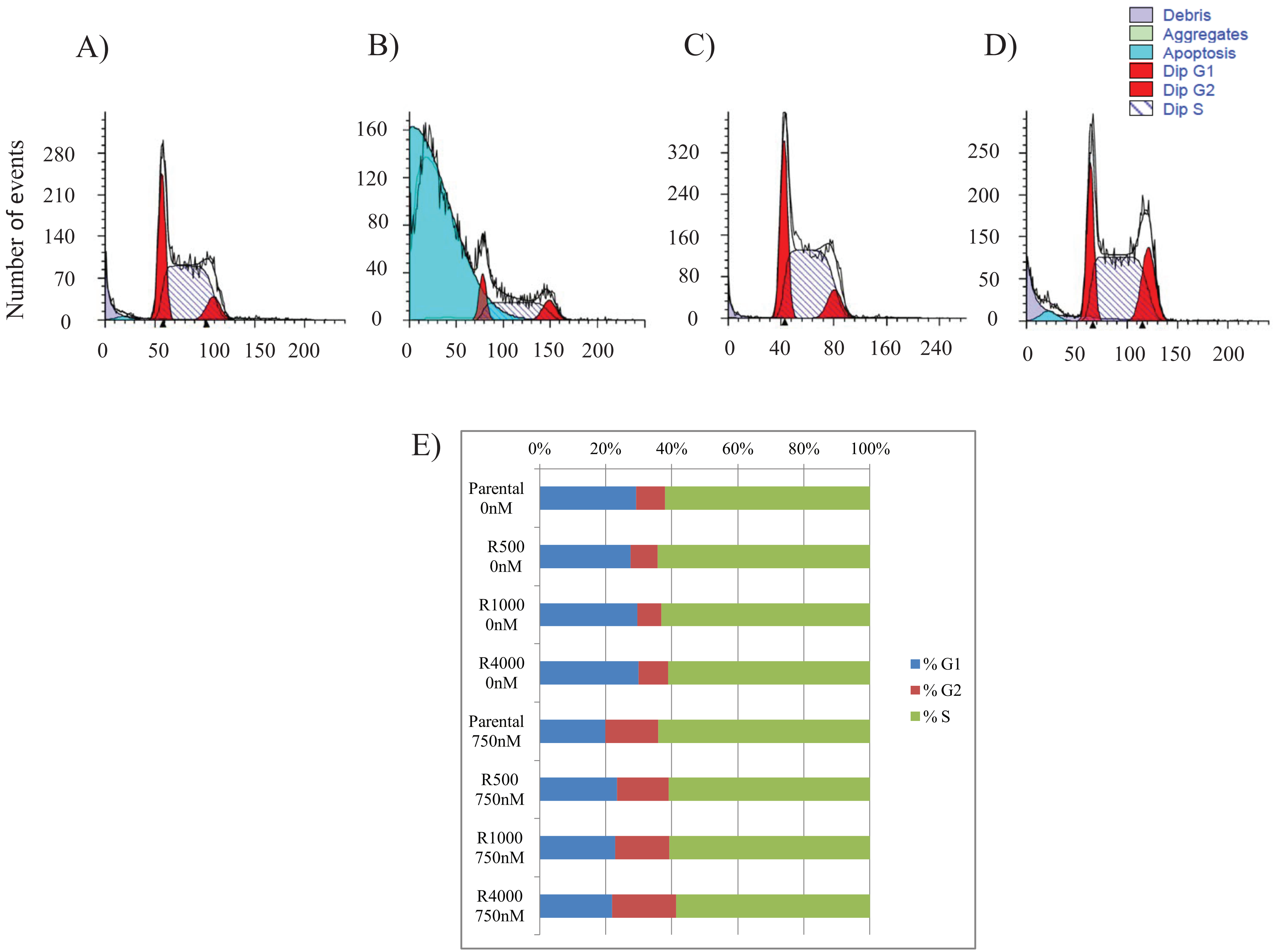

Supplementary Figure 3

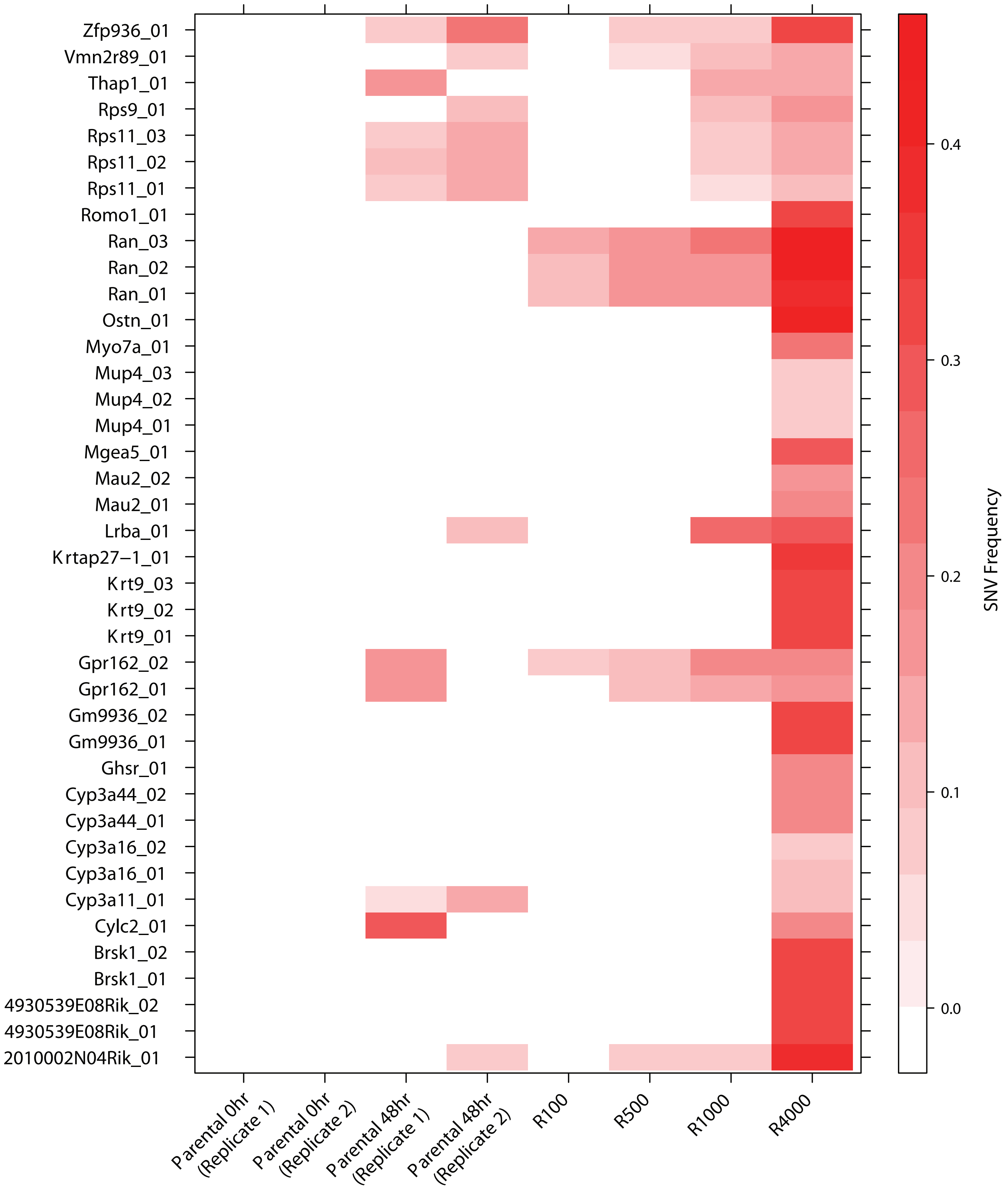

Supplementary Figure 4A

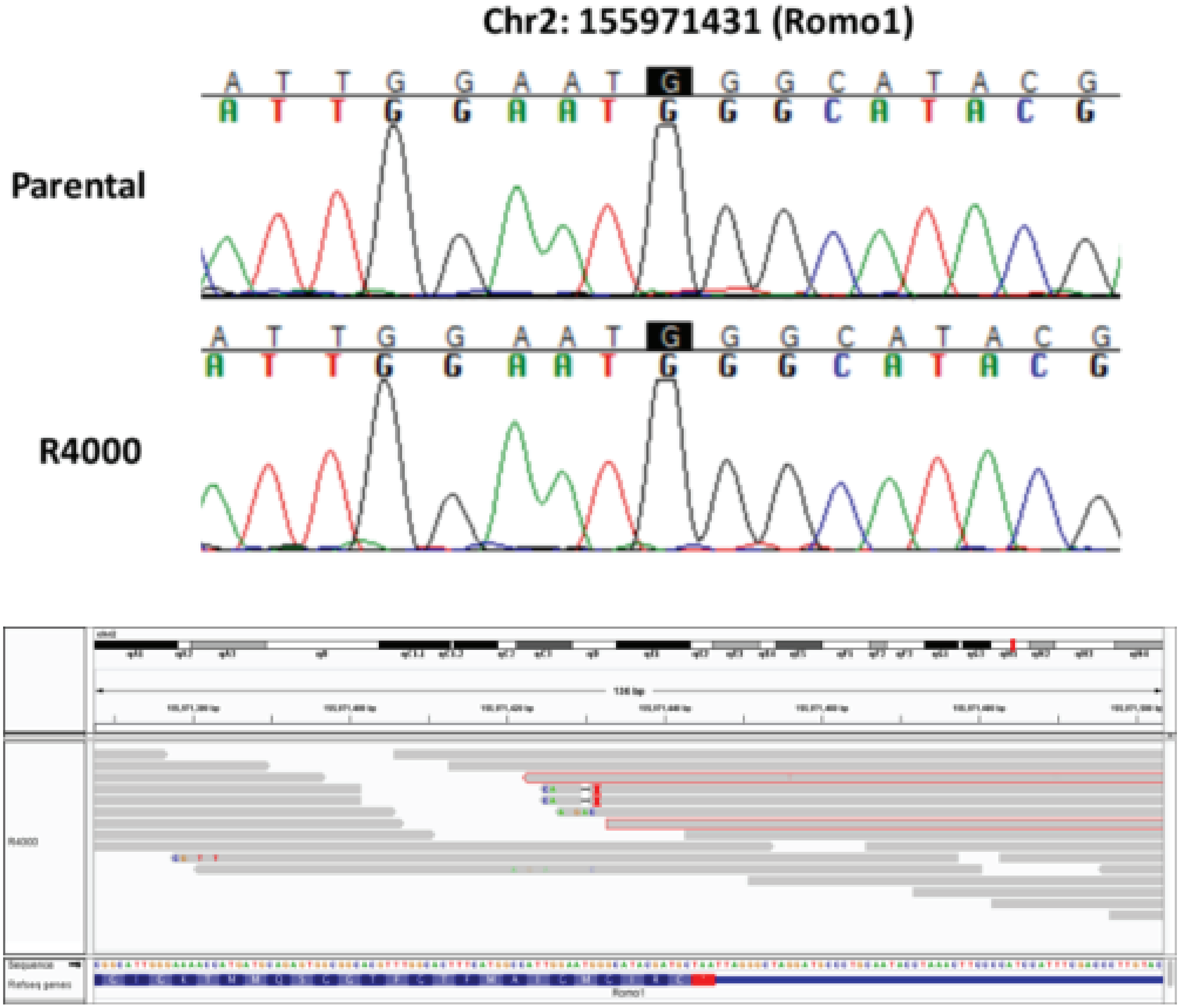

Supplementary Figure 4B

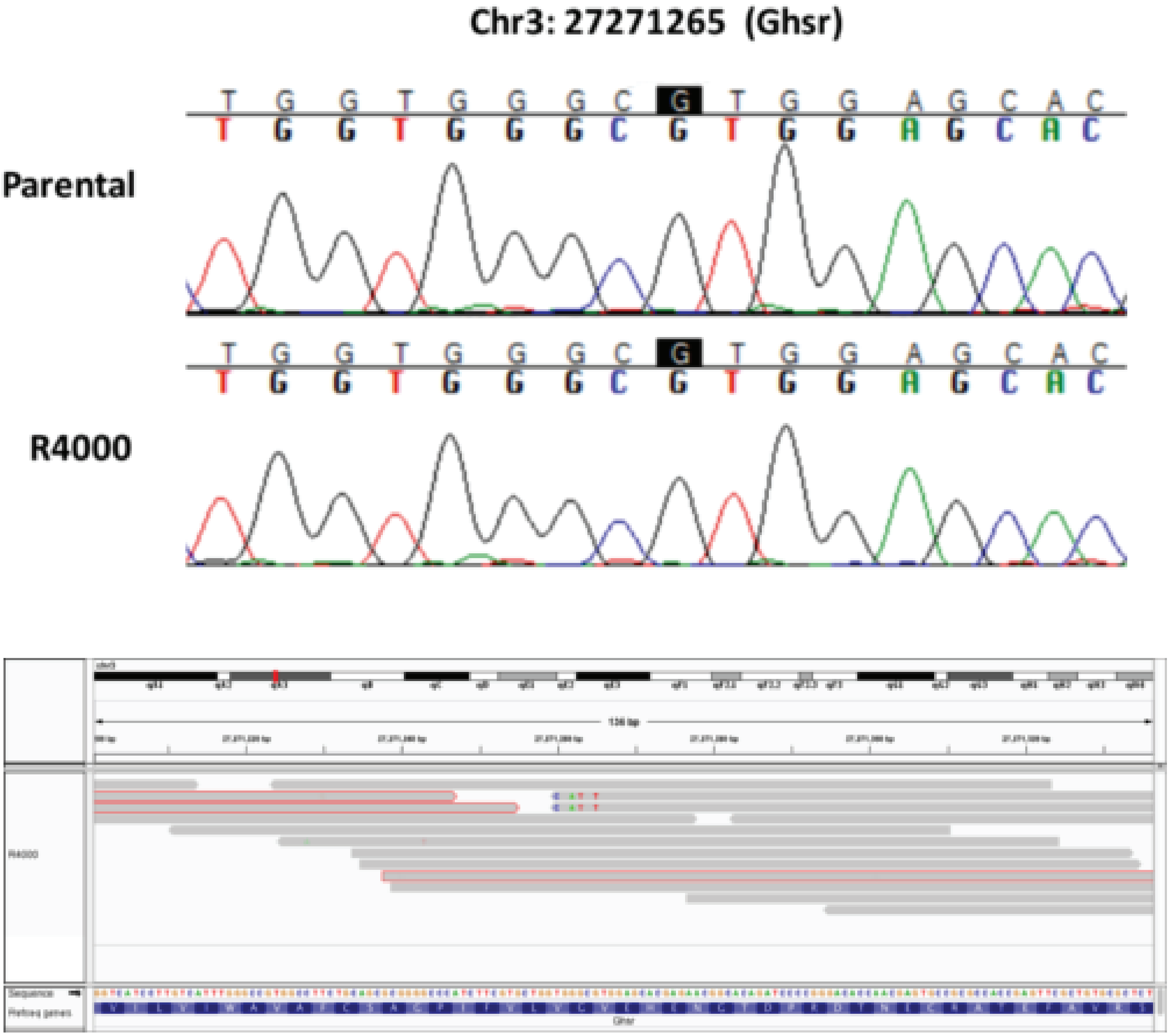

Supplementary Figure 4C

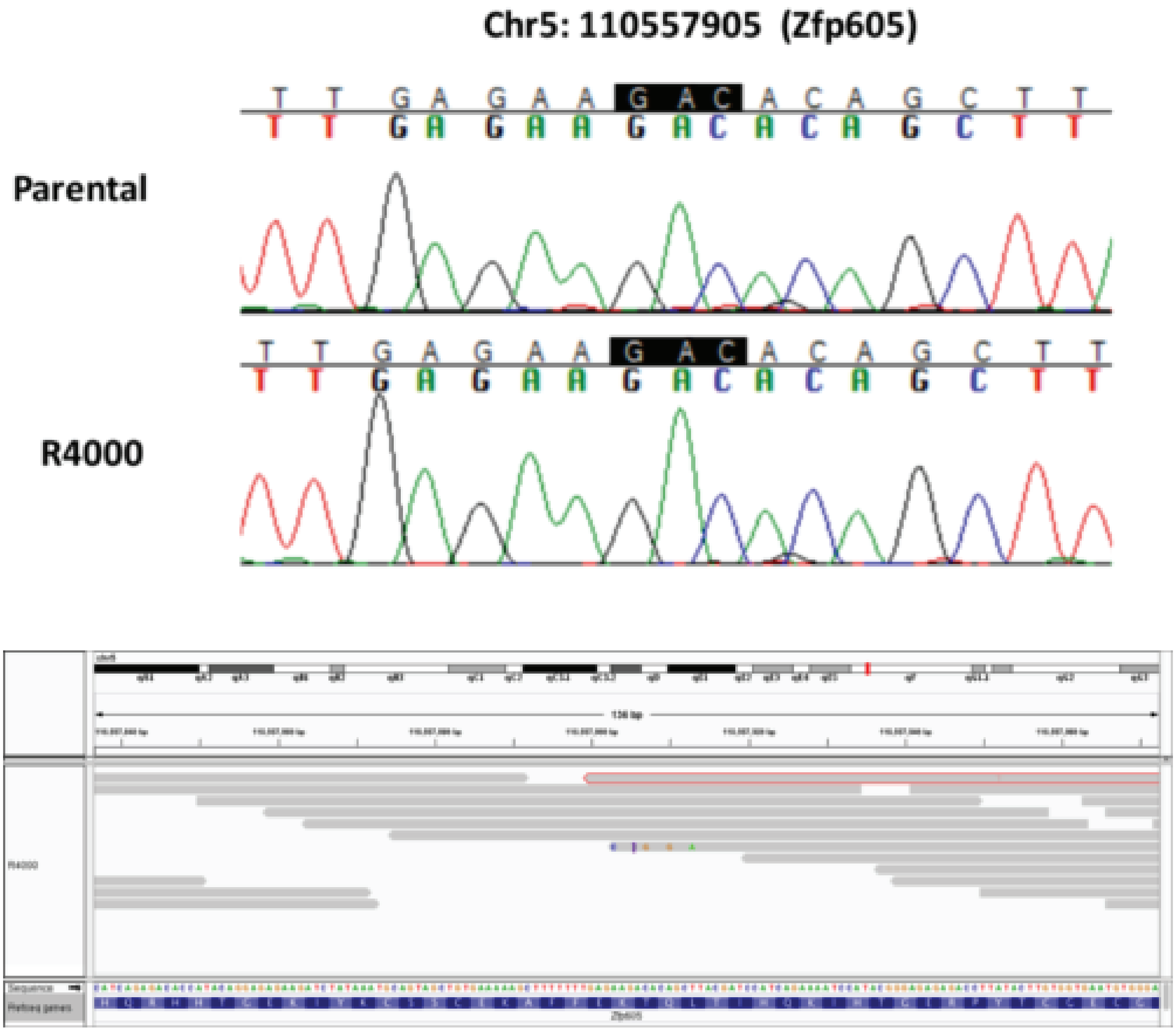

Supplementary Figure 4D

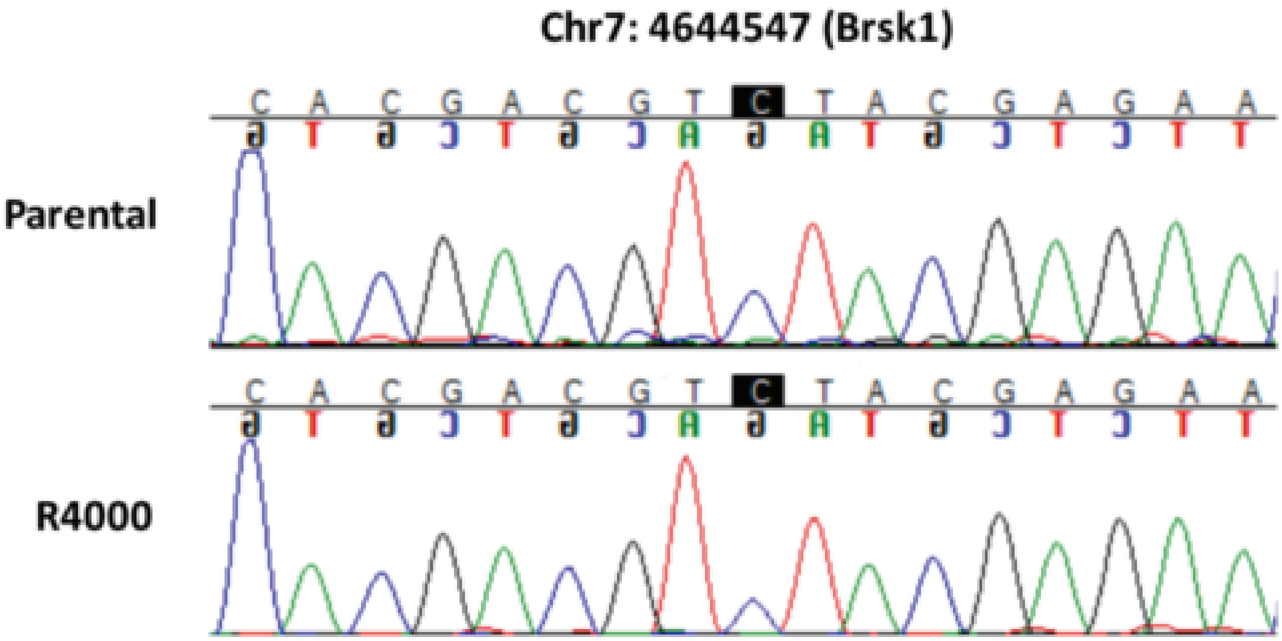

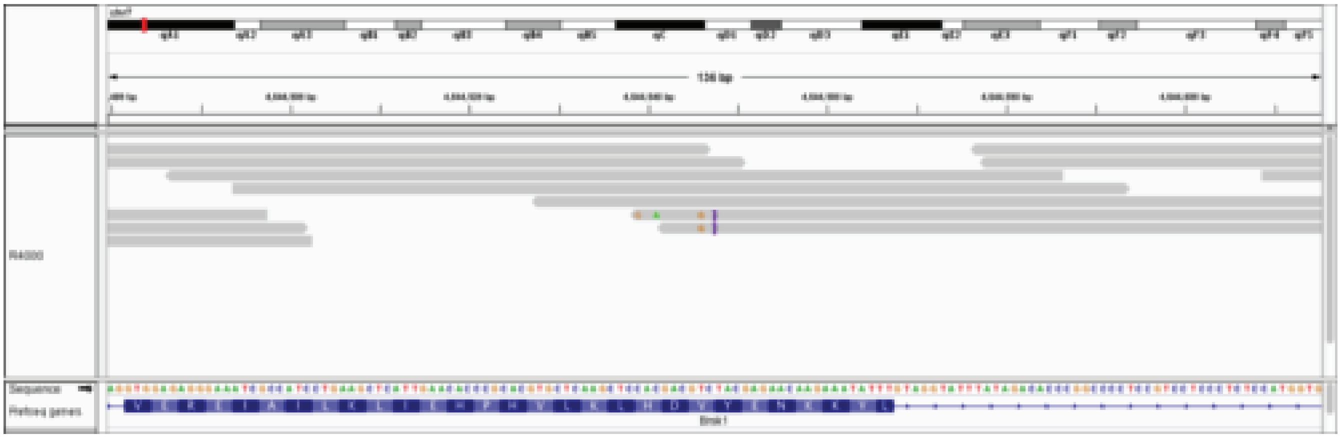

Supplementary Figure 4E

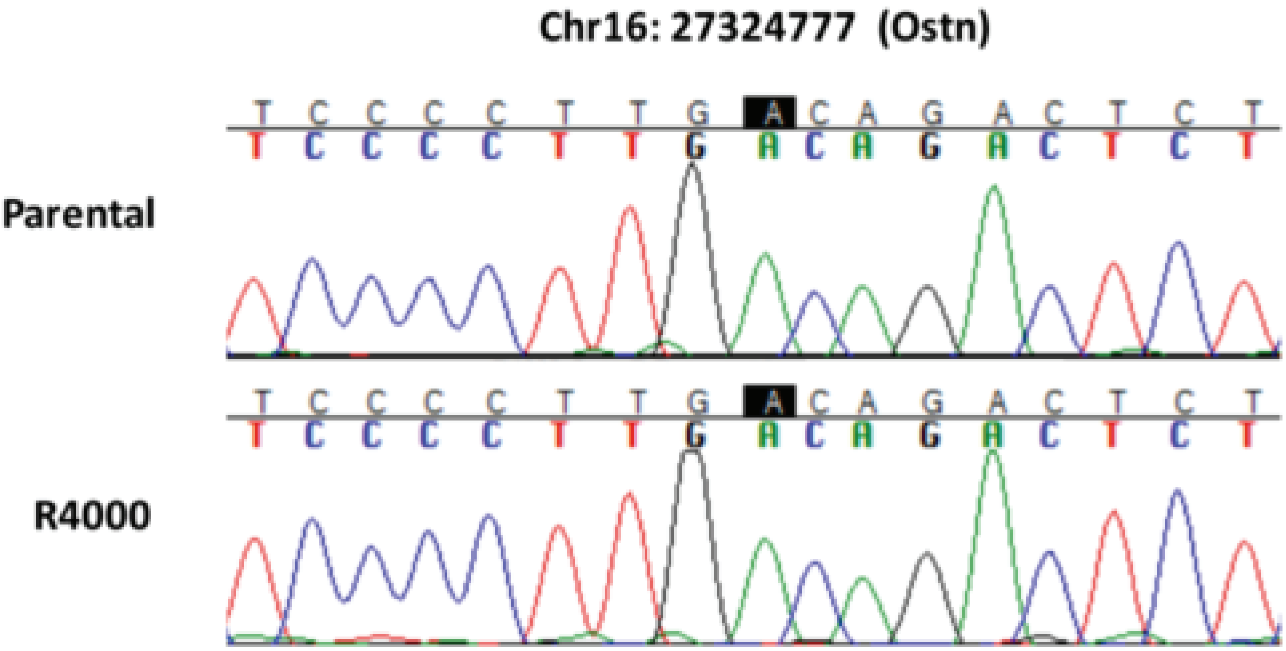

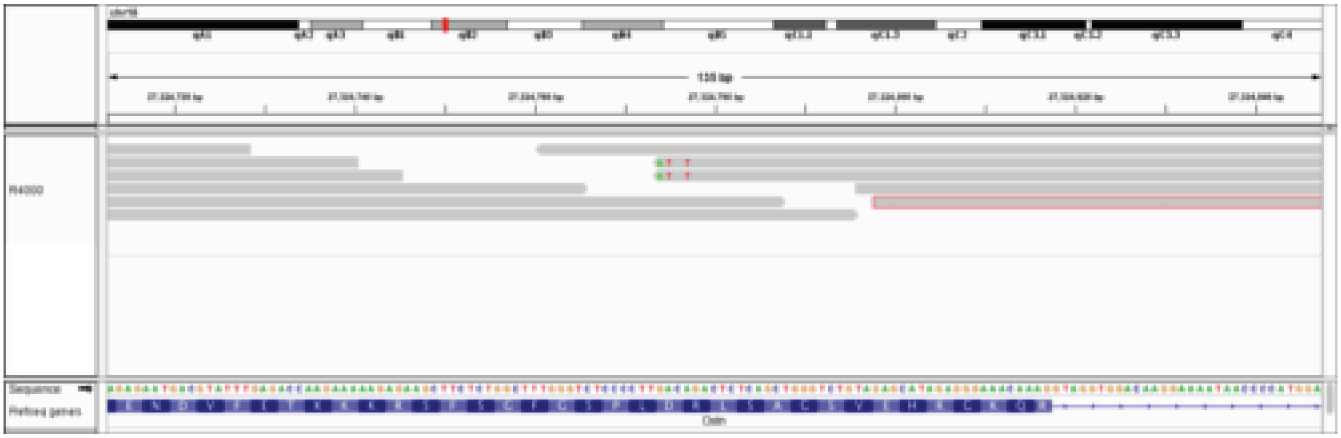

Supplementary Figure 5

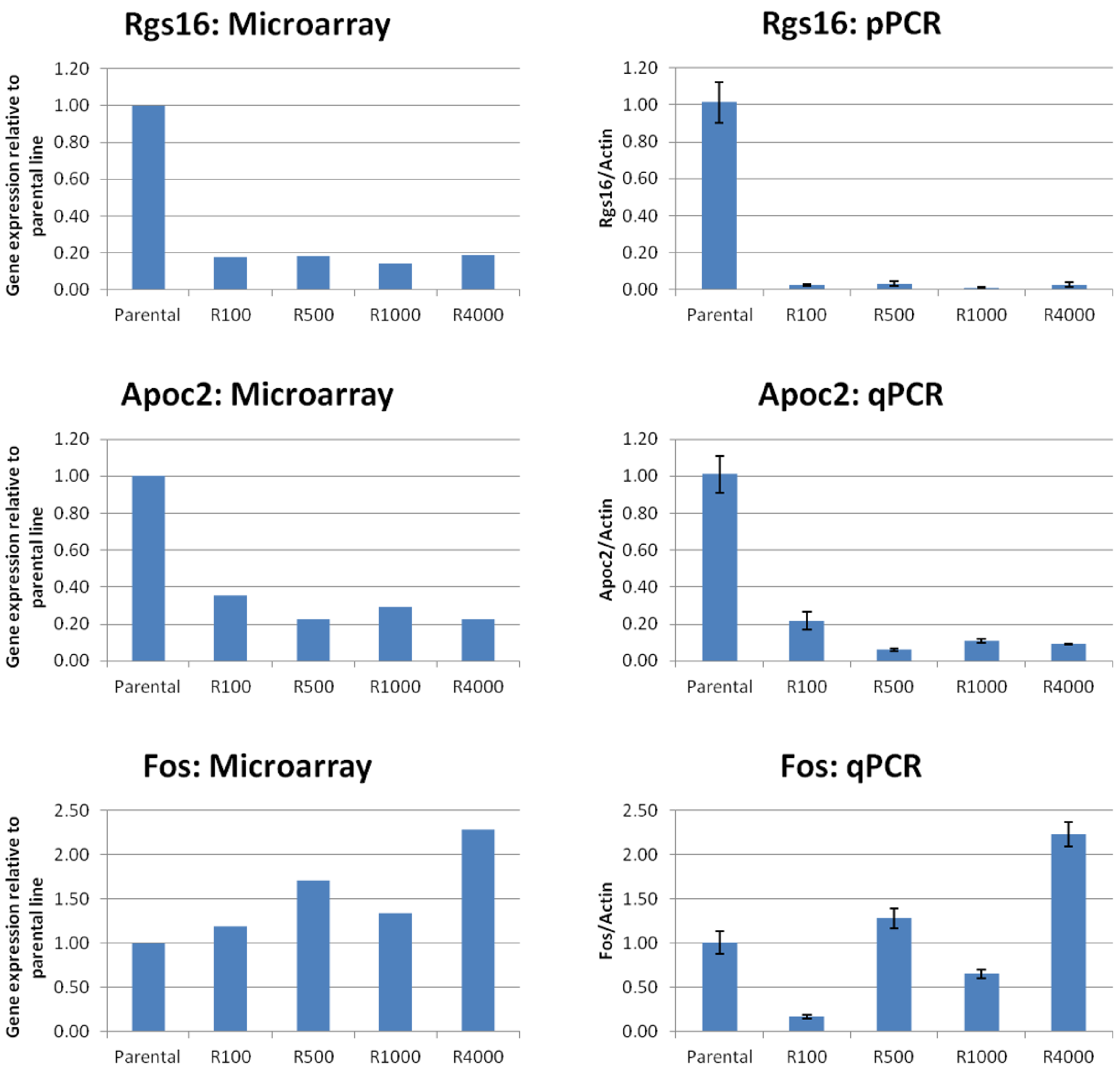

Supplementary Figure 6

A)

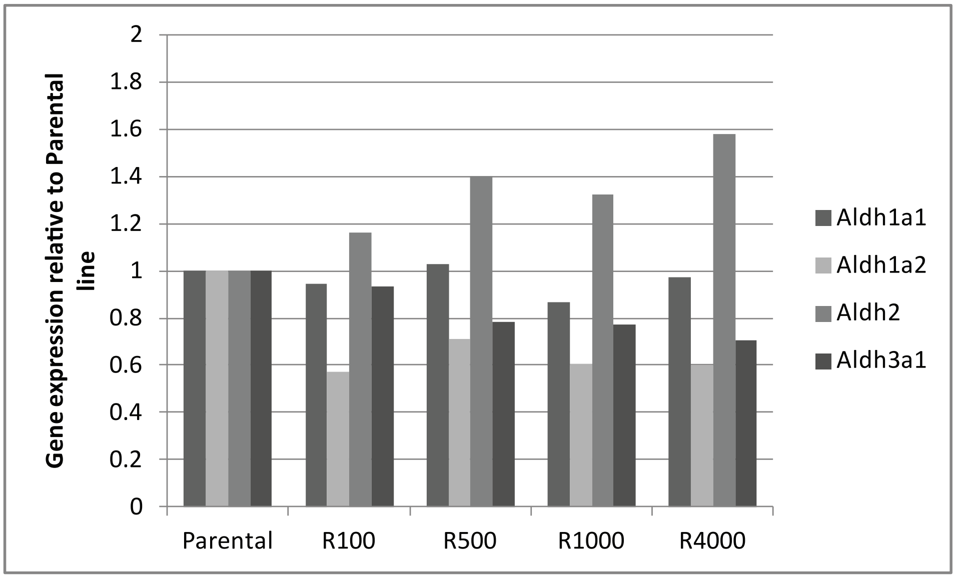

B)

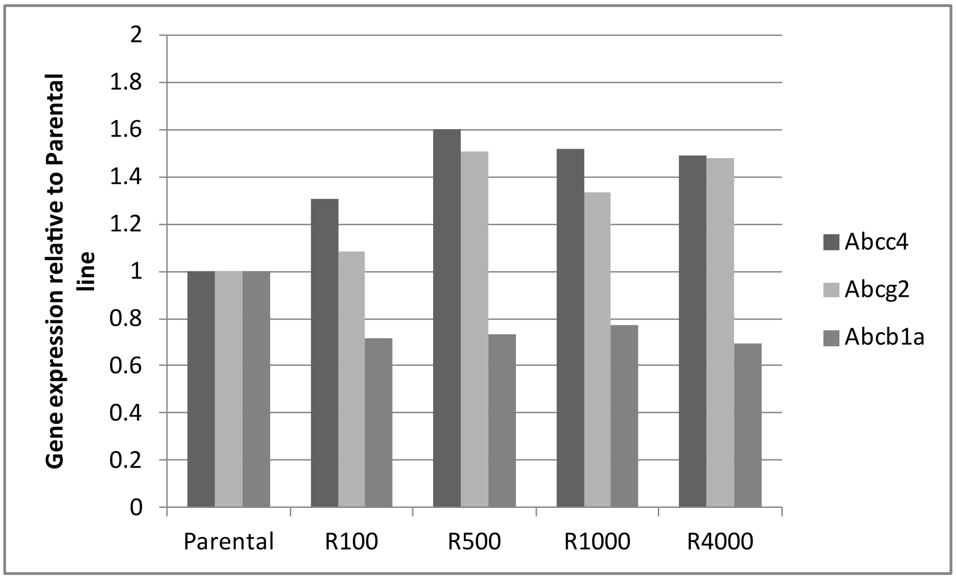

C)

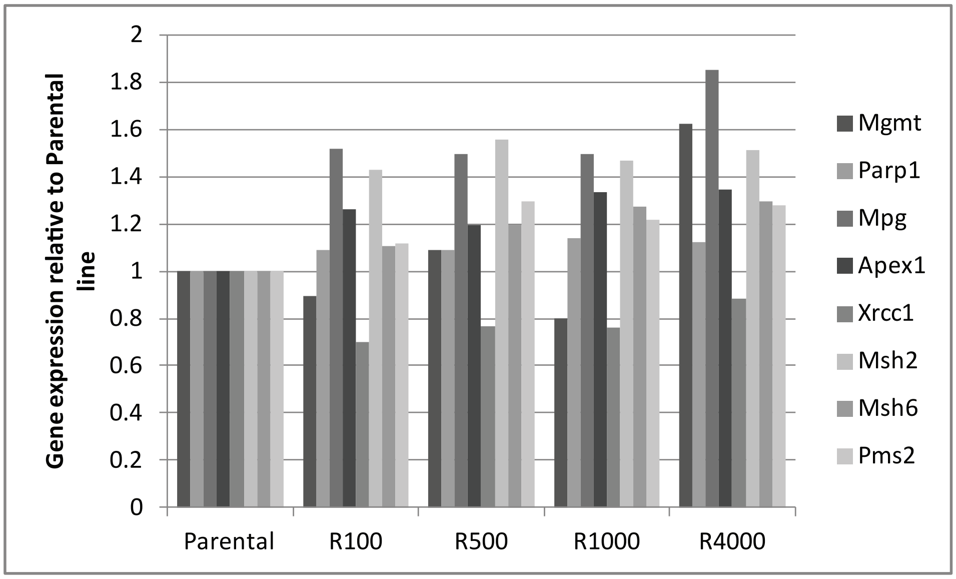

Supplementary Figure 7

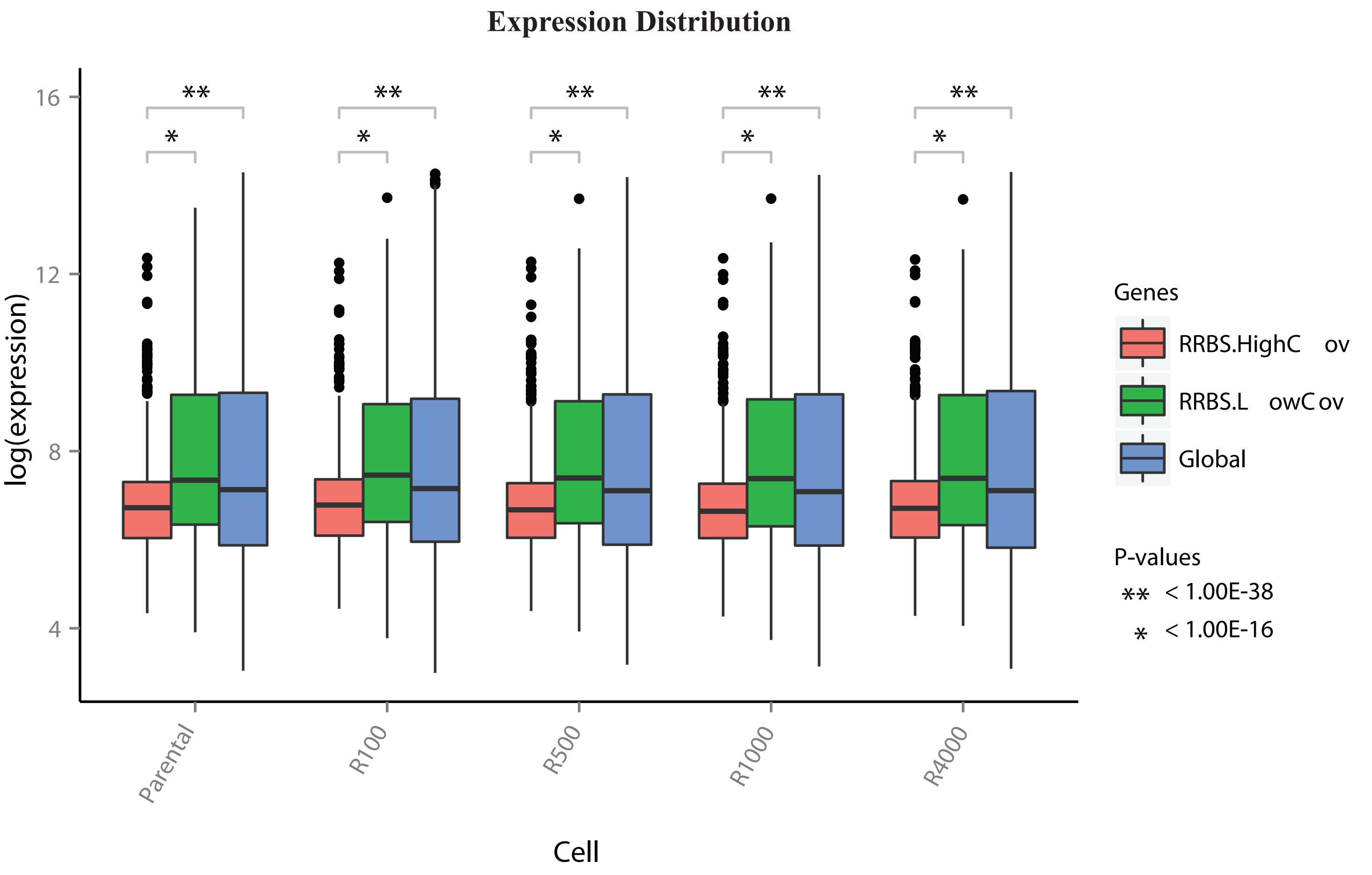

Supplementary Figure 8

A)

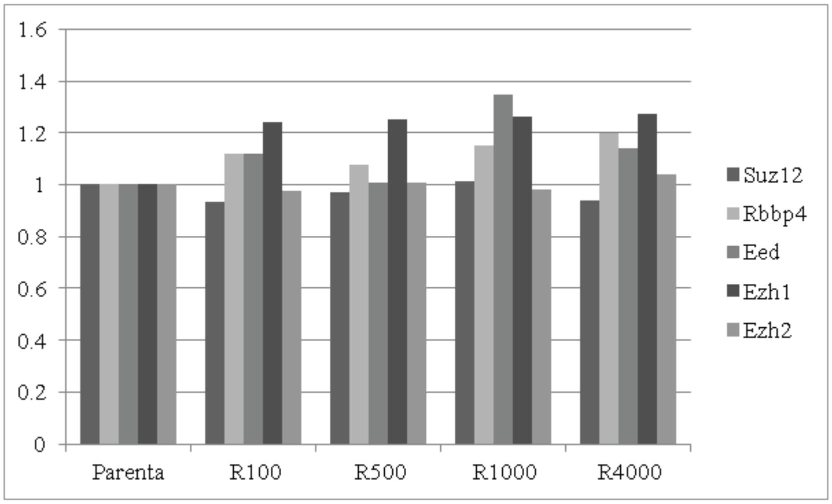

B)

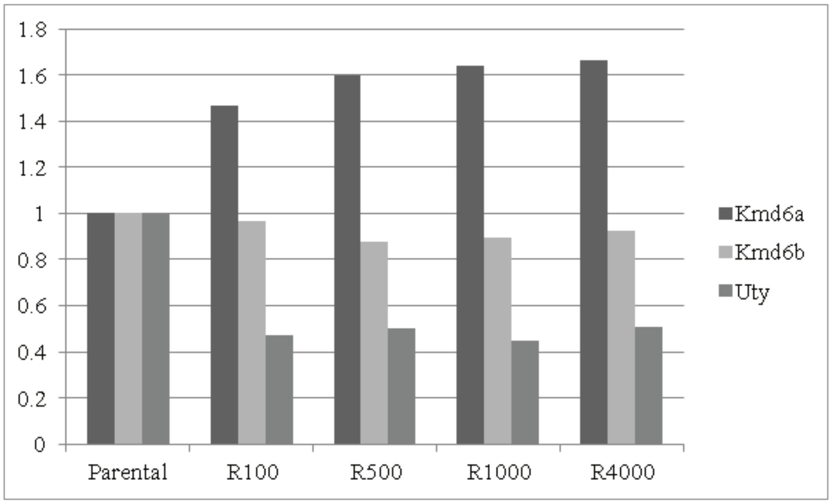

Supplementary Figure 9

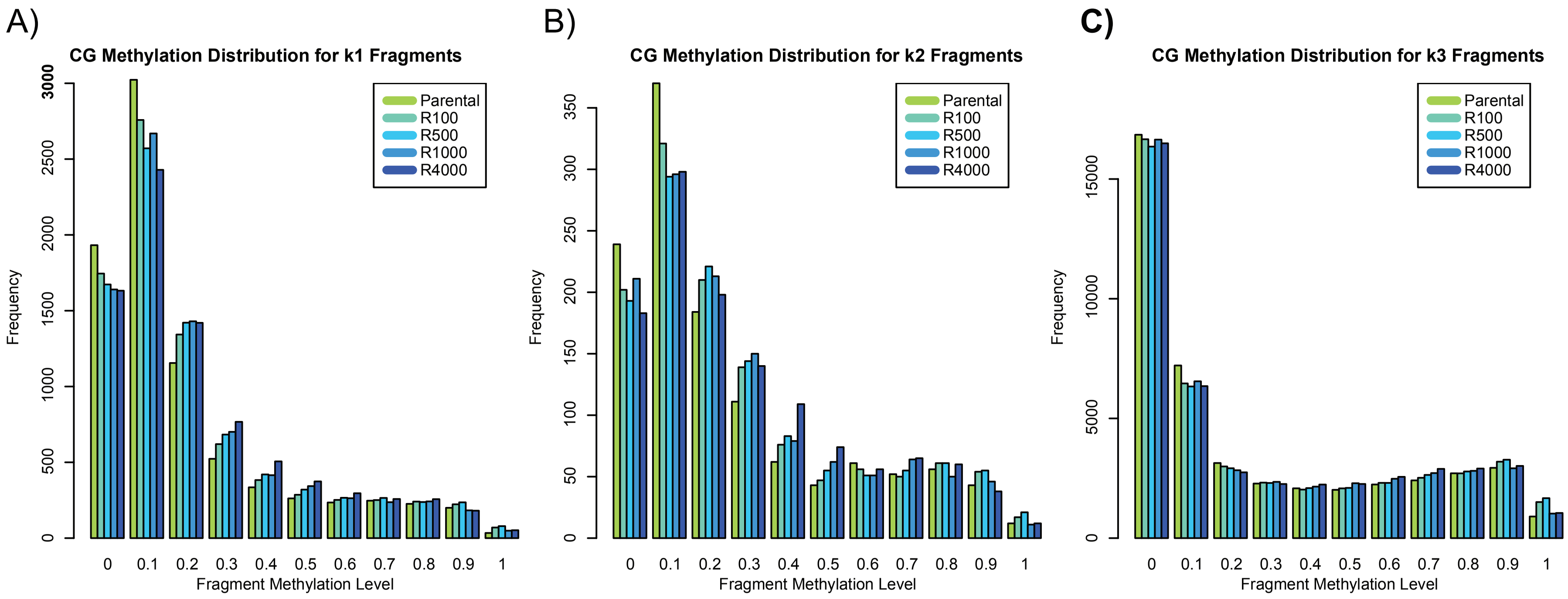

Supplementary Figure 10

A)

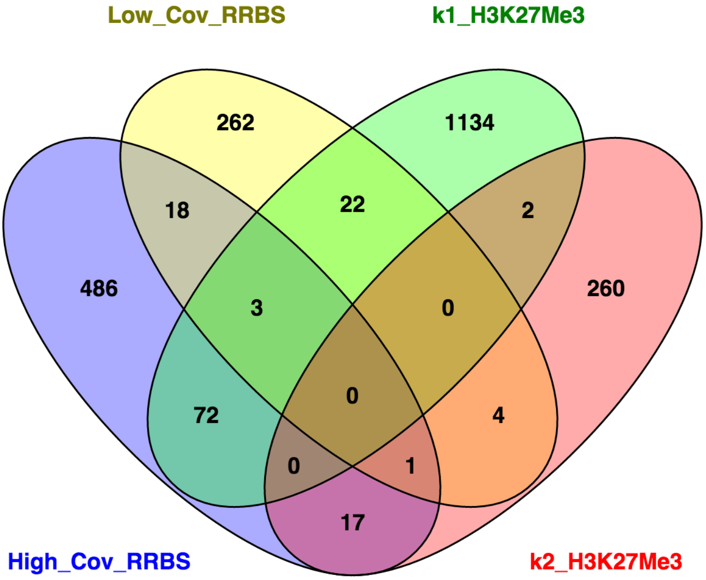

B)

Comparison	# RRBS Genes	# H3K27Me3	# Intersect	p-value
High Cov. & k1	597	1477	75	0.00E+00
High Cov. & k2	597	360	18	5.99E-05
Low Cov. & k1	310	1477	25	1.75E-03
Low Cov. & k2	310	360	5	2.19E-01

Supplementary Figure 11

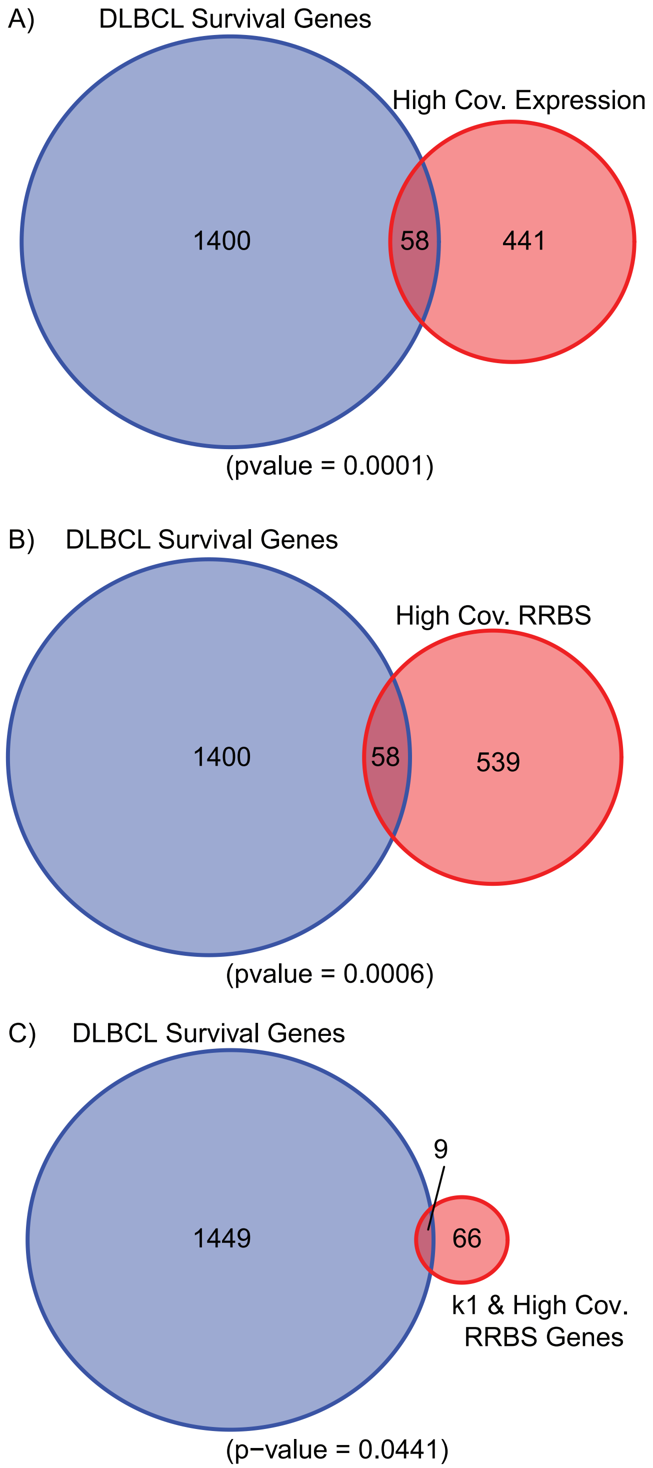

Supplementary Table 1

Cell Line	Treatment	Non-Apoptotic			% Apoptotic
		% G1	% G2	% S	
Eu-Myc Parental	0nM	29.22	8.86	61.92	1.36
Eu-Myc Parental	750nM	21.63	17.57	69.79	81.47
Eu-Myc R500	0nM	27.49	8.25	64.26	0.78
Eu-Myc R500	750nM	23.39	15.72	60.89	46.5
Eu-Myc R1000	0nM	29.62	7.2	63.18	1.03
Eu-Myc R1000	750nM	22.83	16.55	60.55	36.3
Eu-Myc R4000	0nM	29.93	9	61.07	0.1
Eu-Myc R4000	750nM	22.06	19.27	58.67	3.26

Supplemtary Table S2

Comment	Chromosome	Position	Reference	Alternate	Impact	Type	Genes
All ends of reads, likely sequencing artifac	chr2	155971431	G	GCAGAAGAC	High	Frame shift	Rbm39, Romo1, Nfs1
All ends of reads, likely sequencing artifac	chr3	27271265	G	GATCT	High	Frame shift	Ghsr
MMSAT4 satellite repeat	chr5	110557905	GAC	G	High	Frame shift	Zfp605
MMSAT4 satellite repeat	chr5	110557910	A	G	Moderate	Non-synonymous (coding)	Zfp605
End of read for one of two alernate reads	chr5	115307559	C	A	Moderate	Non-synonymous (coding)	4930519G04Rik, Gm9936
End of read for one of two alernate reads	chr5	115307560	C	T	Moderate	Non-synonymous (coding)	4930519G04Rik, Gm9936
Alternate reads for parental line are found at a different copy of this repeat region	chr5	129526821	G	A	Moderate	Non-synonymous (coding)	Ran
Alternate reads for parental line are found at a different copy of this repeat region	chr5	129526836	G	A	Moderate	Non-synonymous (coding)	Ran
Alternate reads for parental line are found at a different copy of this repeat region	chr5	129526848	G	A	Moderate	Non-synonymous (coding)	Ran
Misalignment	chr7	3656339	A	G	Low	Synonymous (coding)	Tsen34, Rps9
Misalignment	chr7	3656351	T	G	Moderate	Non-synonymous (coding)	Tsen34, Rps9
Misalignment	chr7	3656354	C	T	Low	Synonymous (coding)	Tsen34, Rps9
All ends of reads, likely sequencing artifac	chr7	4644546	T	G	Moderate	Non-synonymous (coding)	Brsk1
All ends of reads, likely sequencing artifac	chr7	4644547	C	CA	High	Frame shift	Brsk1

Supplemtary Table S2 (continued)

Satellite repeat region	chr7	50444853	A	G	Moderate	Non-synonymous (coding)	Zfp936
All ends of reads, likely sequencing artifac	chr7	105219534	G	A	Moderate	Non-synonymous (coding)	Myo7a
All ends of reads, likely sequencing artifac	chr7	105219535	C	T	Low	Synonymous (coding)	Myo7a
All ends of reads, likely sequencing artifac	chr11	100054428	C	T	Moderate	Non-synonymous (coding)	Krt9
All ends of reads, likely sequencing artifac	chr11	100054433	T	C	Moderate	Non-synonymous (coding)	Krt9
All ends of reads, likely sequencing artifac	chr11	100054435	C	T	Moderate	Non-synonymous (coding)	Krt9
All ends of reads, likely sequencing artifac	chr16	27324777	A	ATCT	High	Stop gained	Ostn
Mucin tandem repeats	chr16	32755664	T	C	Low	Synonymous (coding)	Muc4
OK	chr16	88671883	C	T	Moderate	Non-synonymous (coding)	Krtap27-1

Supplementary Table 3

Ontology	High Cov. Term Name	Hyper FDR Q-Val	Hyper Foreground Gene Hits	Total Genes Annotated
GO Molecular Function	sequence-specific DNA binding	4.98E-16	92	653
GO Molecular Function	nucleic acid binding transcription factor activity	1.79E-14	103	830
GO Biological Process	cell differentiation	3.26E-25	197	2082
GO Biological Process	anatomical structure morphogenesis	5.08E-23	167	1478
GO Biological Process	nervous system development	7.60E-21	148	1338
GO Cellular Component	transcription factor complex	1.42E-04	34	300
GO Cellular Component	tight junction	7.12E-03	13	96

Supplmentary Table 4

Cell line	Correlation Coefficent
Parental	0.919
R100	0.952
R500	0.948
R1000	0.935
R4000	0.928

Supplementary Table 5

Category	Enriched Terms (PC1 High)	Count	List Total	Pop Hits	Pop Total	FDR
SP_PIR_KEYWORDS	Secreted	101	655	1420	17854	1.79E-07
INTERPRO	Keratin, high sulphur B2 protein	12	643	31	17763	8.66E-06
INTERPRO	Claudin	10	643	28	17763	5.81E-04
GOTERM_CC_FAT	extracellular region part	58	467	774	12504	7.11E-04
**Category**	**Enriched Terms (PC1 Low)**	**Count**	**List Total**	**Pop Hits**	**Pop Total**	**FDR**
GOTERM_CC_FAT	mitochondrion	230	610	1322	12504	2.92E-71
GOTERM_CC_FAT	mitochondrial part	117	610	524	12504	4.47E-43
GOTERM_CC_FAT	ribonucleoprotein complex	102	610	462	12504	2.04E-36
GOTERM_CC_FAT	membrane-enclosed lumen	167	610	1174	12504	3.45E-36
GOTERM_BP_FAT	RNA processing	92	643	437	13588	1.73E-31
GOTERM_BP_FAT	ncRNA metabolic process	63	643	202	13588	9.43E-31
GOTERM_BP_FAT	ncRNA metabolic process	63	643	202	13588	9.43E-31
SP_PIR_KEYWORDS	protein biosynthesis	42	860	139	17854	1.53E-18
GOTERM_CC_FAT	nuclear lumen	112	610	883	12504	2.78E-18
GOTERM_BP_FAT	tRNA metabolic process	36	643	113	13588	1.45E-16
SMART	WD40	34	273	259	9131	1.20E-09
SP_PIR_KEYWORDS	protein transport	53	860	461	17854	1.35E-05
SP_PIR_KEYWORDS	translocation	16	860	65	17854	4.49E-04
**Category**	**Enriched Terms (PC2 High)**	**Count**	**List Total**	**Pop Hits**	**Pop Total**	**FDR**
KEGG_PATHWAY	B cell receptor signaling pathway	20	252	80	5738	9.59E-07
UP_SEQ_FEATURE	Complementarity-determining-3	11	742	23	16021	4.83E-05
GOTERM_MF_FAT	SH3 domain binding	15	590	79	13288	1.23E-02
**Category**	**Enriched Terms (PC2 Low)**	**Count**	**List Total**	**Pop Hits**	**Pop Total**	**FDR**
KEGG_PATHWAY	Ribosome	21	270	89	5738	3.38E-06
GOTERM_CC_FAT	endoplasmic reticulum part	31	571	231	12504	2.93E-04
